# Promiscuity mapping of the S100 protein family using a high-throughput holdup assay

**DOI:** 10.1038/s41598-022-09574-2

**Published:** 2022-04-07

**Authors:** Márton A. Simon, Éva Bartus, Beáta Mag, Eszter Boros, Lea Roszjár, Gergő Gógl, Gilles Travé, Tamás A. Martinek, László Nyitray

**Affiliations:** 1 Department of Biochemistry, Eötvös Loránd University, 1117 Budapest, Hungary; 2 Department of Biochemistry, Semmelweis University, 1094 Budapest, Hungary; 3 Department of Medical Chemistry, University of Szeged, 6720 Szeged, Hungary; 4 MTA-SZTE Biomimetic Systems Research Group, University of Szeged, 6720 Szeged, Hungary; 5 Equipe Labellisee Ligue 2015, Department of Integrated Structural Biology, Institut de Genetique et de Biologie Moleculaire et Cellulaire (IGBMC), INSERM U1258/CNRS UMR 7104/Universite de Strasbourg, 1 rue Laurent Fries, BP 10142, 67404 Illkirch, France

**Keywords:** Biochemistry, Chemical biology, Systems biology

## Abstract

S100 proteins are small, typically homodimeric, vertebrate-specific EF-hand proteins that establish Ca^2+^-dependent protein–protein interactions in the intra- and extracellular environment and are overexpressed in various pathologies. There are about 20 distinct human S100 proteins with numerous potential partner proteins. Here, we used a quantitative holdup assay to measure affinity profiles of most members of the S100 protein family against a library of chemically synthetized foldamers. The profiles allowed us to quantitatively map the binding promiscuity of each member towards the foldamer library. Since the library was designed to systematically contain most binary natural amino acid side chain combinations, the data also provide insight into the promiscuity of each S100 protein towards all potential naturally occurring S100 partners in the human proteome. Such information will be precious for future drug design to interfere with S100 related pathologies.

## Introduction

The vertebrate-specific calcium-binding S100 protein family (termed here as the S100ome) belongs to the superfamily of the EF-hand containing proteins and consists of at least 20 core members of small, usually homodimeric proteins of monomer mass of 10 kDa. These proteins play role in cellular regulation both intra- and extracellularly via protein–protein interactions (PPIs) in a Ca^2+^-dependent manner^1,2^. Under physiological conditions, their expression pattern is tissue-specific and they are present usually in low concentrations^3^. However, their expression level and pattern can be altered under pathological conditions, leading to severe consequences^4^. Specifically, elevated cellular concentrations of certain S100 proteins were observed in cancer, cardiomyopathies, inflammatory and neurodegenerative diseases^4,5^, pointing to them as potential biomarkers and/or therapeutic targets of these diseases^6^. Development of selective inhibitors have great pharmaceutical potential, but it is still challenging due to the structural similarity within the S100 family. Thus, comprehensive, and accurate mapping of the specific S100 interactome is required for such purpose^7^. Although numerous S100 binding partners are known, they are rather restricted to a small subset of the protein family (e.g. S100B, S100A4)^1^. Therefore, a family-wide systematic screening is in need to map the specificity and affinity profiles within the entire S100 family and to identify new binding partners.

**Figure 1:**
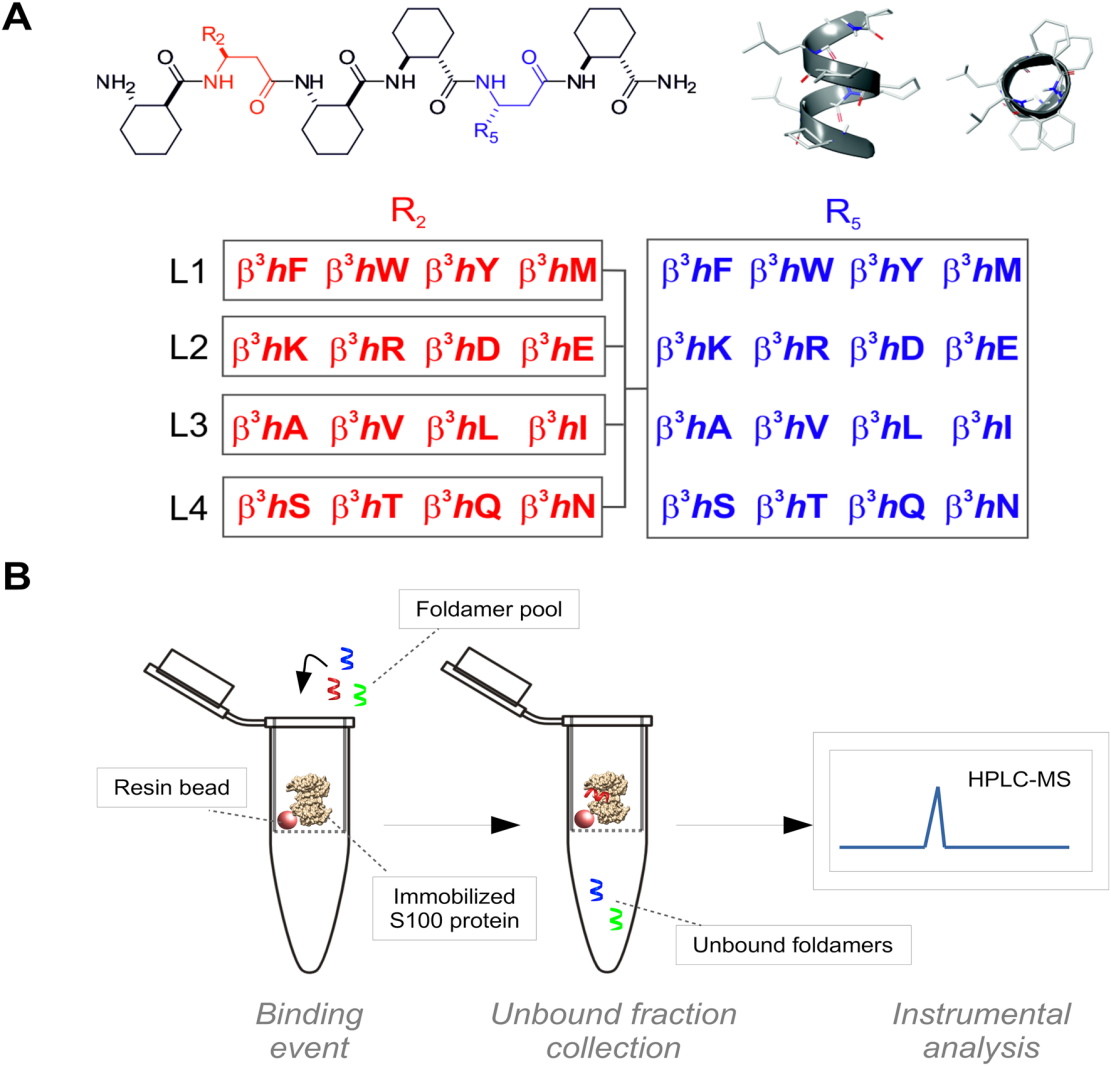
The methodology of the high-throughput (HTP) holdup (HU) assay. (Panel **A**) General sequence of the foldamer library members and structure of H14 helix in side and plan view generated manually by Schrödinger Maestro 11.7 molecular modelling software. The 256-membered foldamer library was divided into 4 sublibraries (L1–L4) based on the general characteristic of the second amino acid (labelled with red) in the sequence. Each sublibrary consists of 64 individuals (R2: four different amino acids, R5: sixteen different amino acids). These four sublibraries (L1–L4) are aromatic, charged, aliphatic, and polar, respectively. (Panel **B**) His-tagged S100 proteins immobilized on Co^2+^-resin (left panel) are incubated with the H14 foldamer library (256 members). The unbound fraction (flow-through) is recovered (middle panel) and the flow-through fractions are analyzed by LC–MS.

Experimental characterization of protein surfaces having shallow binding clefts is a great challenge in drug discovery; however, tools of fragment-based approaches have become efficient techniques toward the identification of small-molecule drug candidates^8^. Mapping the binding surface of proteins can be performed with short recognition elements (i.e., small patches of the binding interface) displaying reduced structural complexity^9–11^. Statistical analyses of protein–protein contact geometries revealed that similar interfaces can be generated by pairs of complexes whose secondary structures are completely different.^12,13^ The consequence of this phenomenon is that the mimicry of a protein–protein interface does not necessarily require the close imitation of the contacting secondary structures. These results suggested a surface fragment approach, in which short foldameric probes were applied for screening shallow binding clefts of protein targets^14^. The foldamer surface fragments are β-peptidic hexamers that fold into a bulky helical conformation in aqueous solution^15^, and present two spatially adjacent proteinogenic side chains toward the target protein surface^16,17^. The short helix cannot mimic an entire protein-helix interface. It behaves as a surface fragment with a tendency to adapt its position, angle and side chain geometry relative to the target surface patch. The stable secondary structure is induced by the conformationally constrained cyclic amino acids (ACHC: *trans*-2-aminocyclohexancarboxylic acid) in the sequence. Our experiments however revealed that the protein recognition is governed by the proteinogenic side chains in positions 2 and 5 (Fig. 1A). The recognition patterns were proteomimetic in terms of surface area specific binding affinity, side-chain enrichment and target specific interactions.^14^ Our goal was to systematically characterize the binding properties of the S100ome with the foldameric surface fragment library in a high-throughput (HTP) experimental setup. Sixteen different proteinogenic side chains were incorporated at positions 2 and 5 of the hexameric probe resulting in a 256-membered foldamer library (Fig. 1A)^14,15^.

The members of the S100 family are often regarded as rather unspecific, promiscuous proteins^18^. Based on our recent study, the S100ome can be divided into two groups, according to binding preference against several natural S100 partners^7^. The partner preferences give a good approximation for the classification of S100 member with multiple partners; nevertheless, the specificity and affinity profile of S100 proteins without a clear binding preference (orphan) are still unknown. Here we reasoned that the binding surface of the S100ome could be mapped extensively by the application of the foldamer-based library containing most natural side chain combination, which cover the general side chain preference of the S100ome by mimicking the complementary binding surface of interacting partners. In this study, we thoroughly investigated the general and unique characteristics of the binding surface of the S100ome by determining the binding affinities of the diverse H14 foldamer library towards the S100 proteins in a HTP holdup (HU) assay^15,19,20^. Our experimental results revealed the binding preferences of not only S100 proteins with multiple known interactions but also S100 members lacking known interaction partners (orphans) in living organisms.

## Results

### Screening the binding affinities of the S100ome against the H14 foldamer library by a HTP HU assay

We screened the binding affinities of the S100ome towards the H14 library by using a HTP HU assay (Fig. 1B), in which the 256-member foldamer library was divided into four sub-libraries (each containing 64 individual foldamer fragments) (Fig. 1A). S100 proteins were immobilized on Co^2+^-resin through their N-terminal His_6_-tag and incubated with the foldamer sublibraries. Experimental conditions were set so that each S100 protein was in equimolar amount (64 µM) with the global concentration of the foldamer sub-library (containing the 64 foldamer fragments in 1 µM), thus all foldameric fragments had the opportunity to bind independently to the protein target, as described previously^15^. After the co-incubation, the unbound foldamers (the flow-through fraction) were separated from the protein-foldamer complexes (resin-bound fraction). Samples were analyzed on LC–MS system, and library members were characterized quantitatively in all samples by their area under the curve (AUC) in the total ion chromatograms (Fig. S1). The AUC value of the appropriate foldameric element in the flow-through fraction was compared to a control sample (comprising all the components of the assay except the immobilized S100 protein) prepared under the same conditions. In this way, we quantified the fraction of each foldamer that was specifically retained on the resin containing immobilized S100 protein. This approach allowed us to determine a bound fraction values (F_B_). We also investigated the solubility of the hydrophobic WW and fWW fragments using light scattering on 600 nm, to exclude the possibility that foldamer fragments are precipitated during experiments^21^. Aggregation was observed at 500 µM for the unlabeled WW and 10 µM for the labeled fWW fragments (Fig. S2). In the assays, the applied concentration of the unlabeled and labeled fragments were 64 µM and 50 nM, respectively; therefore they are assumed to be soluble.

**Figure 2:**
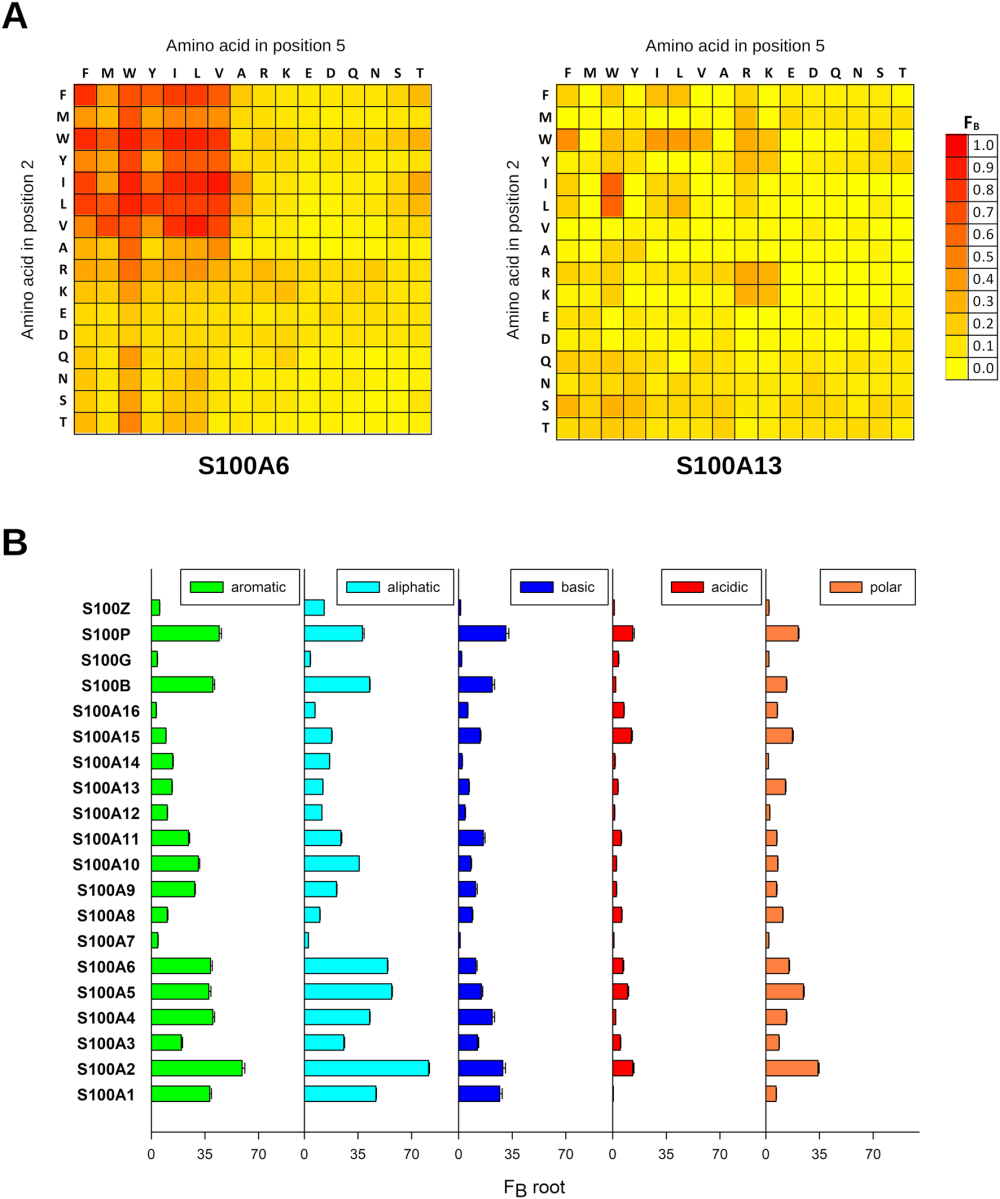
The interaction between the H14 foldamer library and the S100ome measured by holdup (HU) assay. (Panel **A**) The interactions between S100 proteins and foldamers were measured by a high-throughput (HTP) holdup (HU) assay, as visualized in Fig. 1B. Bound fraction values were calculated based on the loss of intensity of the foldamer of interest in the flow-through fraction using Eq. (1) (see “Methods”); and were depicted as a heat map in linear scale for each S100 protein. F_B_ ranges are color coded as shown on the right. The vertical axis and horizontal axis represents the β-amino acid in the second and fifth positions, respectively^14^. Some S100 members favored multiple fragments (e.g. S100A6 on the left), while multiple S100 proteins did not show clear binding preference towards the foldamer fragments (e.g. S100A13 on the right). (Panel **B**) S100 proteins exert different amino acid sidechain preference based on the HTP HU measurements. The amino acid preferences were calculated for all S100 proteins using Eqs. (3) and (4) (see “Methods”). SEM was calculated from the three individual F_B_^root^ values. Mean ± SEM were depicted as a bar chart. The residues with high frequency in the bound foldamers have hydrophobic properties as aromatic and aliphatic side chains are the most preferred ones. Importantly, due to the rather acidic nature of S100 proteins, acidic side chains are the least preferred among S100 proteins. It is noteworthy that in some instances polar residues are also favored (e.g. S100A2, S100A5).

We used this approach to map the binding affinities of the complete S100ome and determined the F_B_ constants of 5120 interactions (20 S100 proteins versus the 256-member foldamer library), depicted as heat maps (Fig. 2A, Fig. S3). The binding patterns of the S100 proteins for foldamer fragments were found to be highly diverse. Some S100 members (e.g. S100A16, S100G) displayed only weak interactions (F_B_ < 0.2) toward the foldamers, while other family members (e.g. S100A2, S100A6) showed high propensity to bind foldamer probes (Fig. S3). The pair of identical hydrophobic binding pockets in S100 homodimers^2^, created by the Ca^2+^-induced conformational changes, could be recognized by highly hydrophobic side chains with limited selectivity and the H14 library generally displayed enrichment for residues Trp, Phe, Ile and Leu. Beside the most favored hydrophobic side chains, which can often be observed in systematic libraries^22^, foldamers containing basic and polar residues were also enriched on the protein binding sites in some cases, providing useful information to increase selectivity in rational drug design (Fig. 2B). S100 family members are rather acidic proteins (pI_average_ = 5.68 ± 0.92), therefore, basic residues (Arg and Lys) are preferred in their ligands over acidic side chains (Glu and Asp). The enrichments of positively charged residues were found significant in our assay for S100A1, S100A2, S100B and S100P; as these S100 family members possess the lowest theoretical pIs (4.39, 4.68, 4.52 and 4.75, respectively). It is notable that neutral polar side chains were also found preferable for some of these family members (e.g. S100A2, S100P).

The binding pattern mostly displayed a diagonal symmetry indicating a closely neutral nature of the template backbone. For some cases, the lack of the symmetric characteristics (e.g. S100A9, S100P) was observed suggesting that the two β^3^-amino acids are not interchangeable with each other, since not only the relative position of the side chains is important, but also the position of the preferred proteinogenic side chains related to the terminals.

### Investigating the interactions between the S100ome and the selected foldamers by fluorescence polarization

**Figure 3:**
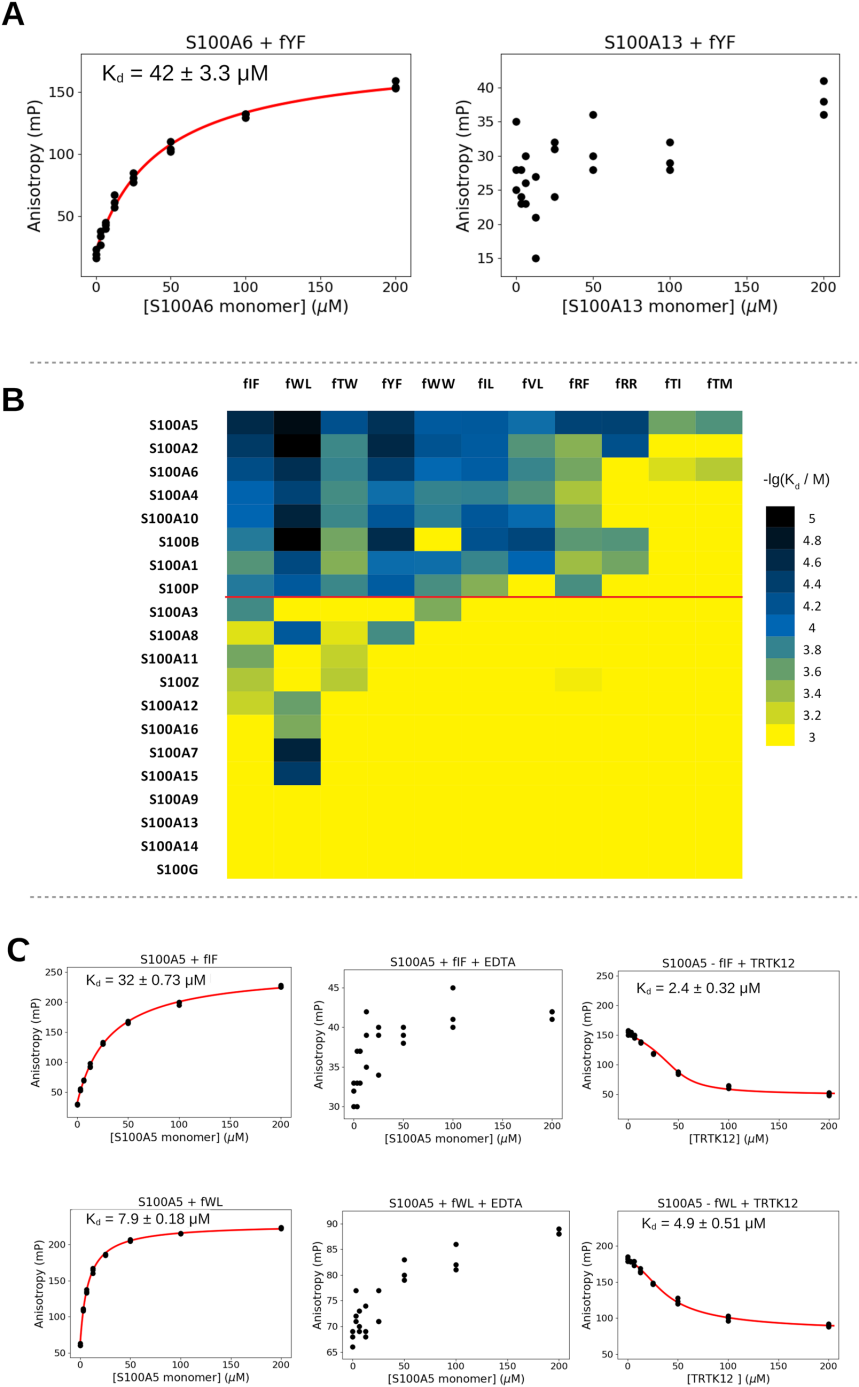
The interactions between the selected foldamers and S100 proteins measured by direct fluorescence polarization (FP). (Panel **A**) The interactions between the S100ome and the labeled foldamer molecules were monitored by direct fluorescence polarization assay, in which the increase of the polarization (i.e. decrease of the rotation) caused by adding S100 proteins is indicative of the binding event, i.e. the association of the labeled foldamer – S100 complex is monitored. Dissociation constants were calculated by fitting the anisotropy values (mP) using quadratic equation with the ProFit program^7^. The dissociation constants were given as mean ± SEM. Left panel: S100A6 was added in various concentrations to the fluorescently labeled foldamer (fYF) and a significant binding event is observed. Right panel: S100A13 was added to the same foldamer and only a minor linear increase of the polarization was noticed confirming the results obtained by the HTP HU assays. (Panel **B**) The –lg(K_d_) values of the interactions between the selected foldamers and the S100ome were depicted as a heat map. –lg(K_d_) ranges are color coded as shown on the right. The specificity-map of the S100ome towards the H14 library correlate well qualitatively with the results of the HTP HU measurements; i.e. S100 proteins (e.g. S100A2, S100A5, S100A6) interacting with numerous foldamer fragments in the HU assays exhibit the same behavior in direct FP measurements, meanwhile S100 members (e.g. S100G, S100A9, S100A13) imposing fewer interaction with the H14 library in the HU assays form weak, or no bound with the selected foldamers. It is noteworthy that based on the specificity map, the S100 proteins can be divided into two groups; one with numerous detected partners (upper part) and one with few or no detected partners (lower part). (Panel **C**) Foldamer fragments bind to the hydrophobic binding pocket of S100 proteins in a calcium-dependent manner. Left panels: examples of direct titration of fIF and fWL in the presence of Ca^2+^ with S100A5, respectively, showing significant binding. Middle panels: the same titrations in the presence of EDTA resulted in the loss of binding event for both foldamer fragments, providing evidence that the S100-foldamer interactions are calcium-dependent. Right panels: Titrating the preformed S100—labeled foldamer complex with an S100 binding peptide, TRTK12, competition between the labeled foldamer fragment and the unlabeled S100-binding peptide is observed in both cases, providing evidence that the foldamer fragments bind to the binding pocket of S100 proteins. K_d_ values were calculates as in Panel **A** using quadratic (left panels) and competitive binding equation (right panels), respectively. All the dissociation constants were given as mean ± SEM.

HTP (and also low-throughput) measurements generally need to be validated by an orthogonal approach to eliminate experimental artifacts^23^. From the H14 library we selected foldamer fragments to analyze in greater depth with relatively high affinity (based on the HTP-HU assays) and different chemical properties (aromatic, aliphatic, polar, acidic, basic) of the corresponding proteogenic side chain. This way, we chose WL, IF, WW, YF, IL, VL, TW, RF, RR, TI, TM, and after resynthesizing with a fluorescent label at the C-terminus (Figs. S15–S26, Table S1), the S100ome was tested against the labeled foldamers by direct fluorescence polarization (FP) (Fig. 3A). In this assay, the association of the fluorescently labeled foldamer and the S100 protein of interest is monitored, through the change in polarization of the emitted light by the fluorophore upon the binding event. In direct FP, the presence of the fluorophore might change the binding affinity of some foldamers. While it would have been preferable to address the binding capacity of non-labelled foldamers by competitive FP^7^, the limited solubility of the compounds and their low affinity did not allow us to set up a competitive assay. Nevertheless, we assumed that the fluorophore would affect all foldamers that target the same binding site to the same degree, because the foldamer scaffold is rigid. Defining the threshold of detection at the dissociation constant of 1 mM, we identified 87 interactions between the selected foldamer fragments and the S100ome out of 220 possible interactions (Table 1, Fig. S4-14).

**Table 1:** The dissociation constants given as mean ± SEM of the selected foldamers and the S100ome measured by direct fluorescence polarization.

	Kd (μM)										
	fIF	fRF	fTM	fTW	fWW	fTI	fIL	fRR	fVL	fWL	fYF
S100A1	210 ± 13	420 ± 81	> 1000	330 ± 20	110 ± 19	> 1000	160 ± 24	280 ± 38	97 ± 12	53 ± 5.2	110 ± 9.8
S100A2	38 ± 1.5	340 ± 21	> 1000	170 ± 5.5	65 ± 1.2	> 1000	78 ± 8.7	61 ± 5.5	210 ± 110	11 ± 0.39	26 ± 2.1
S100A3	180 ± 8.4	> 1000	> 1000	> 1000	310 ± 21	> 1000	> 1000	> 1000	> 1000	> 1000	> 1000
S100A4	93 ± 2.4	460 ± 23	> 1000	180 ± 46	160 ± 9.8	> 1000	150 ± 4.8	> 1000	210 ± 8.7	46 ± 1.6	110 ± 5.2
S100A5	27 ± 0.53	46 ± 1.2	200 ± 9.2	62 ± 1.4	77 ± 2.8	270 ± 14	75 ± 3.6	45 ± 0.81	110 ± 5.4	14 ± 0.55	32 ± 1.0
S100A6	57 ± 1.8	290 ± 14	520 ± 57	160 ± 8.1	100 ± 3.2	700 ± 63	82 ± 5.4	> 1000	170 ± 13	30 ± 1.7	42 ± 3.3
S100A7	> 1000	> 1000	> 1000	> 1000	> 1000	> 1000	> 1000	> 1000	> 1000	23 ± 1.5	> 1000
S100A8	750 ± 46	> 1000	> 1000	770 ± 180	> 1000	> 1000	> 1000	> 1000	> 1000	74 ± 3.5	180 ± 42
S100A9	> 1000	> 1000	> 1000	> 1000	> 1000	> 1000	> 1000	> 1000	> 1000	> 1000	> 1000
S100A10	97 ± 2.5	320 ± 19	> 1000	170 ± 5.6	150 ± 5.2	> 1000	72 ± 1.6	> 1000	110 ± 5.5	23 ± 0.7	70 ± 3.5
S100A11	280 ± 15	1000 ± 220	> 1000	570 ± 68	> 1000	> 1000	> 1000	> 1000	> 1000	> 1000	> 1000
S100A12	590 ± 39	> 1000	> 1000	> 1000	> 1000	> 1000	> 1000	> 1000	> 1000	250 ± 110	> 1000
S100A13	> 1000	> 1000	> 1000	> 1000	> 1000	> 1000	> 1000	> 1000	> 1000	> 1000	> 1000
S100A14	> 1000	> 1000	> 1000	> 1000	> 1000	> 1000	> 1000	> 1000	> 1000	> 1000	> 1000
S100A15	> 1000	> 1000	> 1000	> 1000	> 1000	> 1000	> 1000	> 1000	> 1000	39 ± 2.4	> 1000
S100A16	> 1000	> 1000	> 1000	> 1000	> 1000	> 1000	> 1000	> 1000	> 1000	300 ± 130	> 1000
S100B	140 ± 7.9	230 ± 5.9	> 1000	280 ± 17	> 1000	> 1000	63 ± 4.4	210 ± 26	50 ± 6.4	11 ± 0.81	28 ± 1.7
S100G	> 1000	> 1000	> 1000	> 1000	> 1000	> 1000	> 1000	> 1000	> 1000	> 1000	> 1000
S100P	140 ± 5.8	190 ± 17	> 1000	170 ± 9.8	190 ± 6.9	> 1000	330 ± 92	> 1000	> 1000	75 ± 5.8	79 ± 5.7
S100Z	480 ± 31	890 ± 120	> 1000	520 ± 59	> 1000	> 1000	> 1000	> 1000	> 1000	> 1000	> 1000

The affinity profile of the S100ome was depicted as a heat map (Fig. 3B), using the K_d_ values determined in direct FP measurements fitted with a quadratic binding equation by the ProFit program^7^. Based on the affinities, the S100ome can be divided into two groups. The upper group shown in Fig. 3B contains S100 proteins (S100A5, S100A2, S100A6, S100A4, S100A10, S100B, S100A1, S100P) with multiple detected interactions, which can be characterized by micromolar binding affinities. Meanwhile, the lower part of the heat map consists of members (S100A3, S100A14, S100A8, S100A11, S100Z, S100A13, S100A12, S100A16, S100A7, S100A15, S100G, S100A9) without a clear binding preference implying only a few or no partners amongst the selected foldamers. The arrangement of the S100ome shows a similar pattern compared to our recent study^7^ with the S100ome using natural binding partners.

To ensure that foldamer fragments bind to the hydrophobic binding groove of S100 proteins, which opens upon binding of calcium ions, we performed FP experiments on a selected S100 protein, S100A5, in the presence of EDTA and TRTK12, an S100-binding peptide of 12 amino acids. Our results showed that S100A5 is unable to interact with either fIF or fWL in the absence of Ca^2+^. Moreover, TRTK12 peptide competed with both foldamers with a K_d_ value like our previous results (Fig. 3C)^7^. Based on the similarity and redundancy among the S100 family, it can be assumed that all S100 proteins bind the members of the H14 foldamer library through their hydrophobic binding pocket in a Ca^2+^-dependent manner.

### Mapping promiscuity in the S100ome

Assuming that the H14 library contains all the relevant binary combinations of amino acid side chains covering all the side chain preferences of S100 proteins, our data provide information about the binding promiscuity of each S100 member. Herein, we will refer to "promiscuity" as a parameter capturing both the broadness of exploration of the potential ligand space and the strength of binding to the recognized ligands. Based on the F_B_ values of the HU assays, we define a quantitative promiscuity term that is calculated for each S100 protein by dividing its average bound fraction value against the library by the maximal F_B_ value (Eq. 1). As the theoretical maximum of F_B_ is unity (F_B_ is a value between 0 and 1), promiscuity is equal to the average bound fraction. This way, interacting with only one foldamer results in a low average F_B_ value, however, by binding to multiple members of the foldamer library in a similar manner leads to a higher value of promiscuity parameter.

1 $$P\left(S100\right)= \frac{\overline{{F }_{B}}(S100)}{{F}_{B max}} = \overline{{F }_{B}}(S100)$$

**Figure 4:**
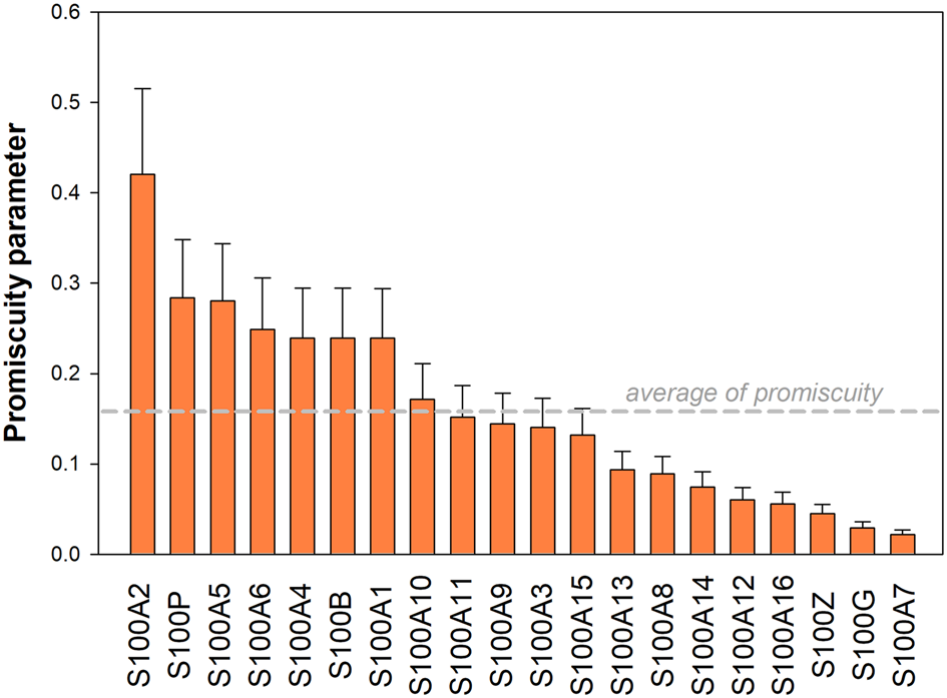
Promiscuity of the S100ome towards the H14 foldamer library. Promiscuity values of the S100ome are defined towards the full H14 foldamer library (all the 256 possible combinations of 16 amino acids in two residues per foldamer building blocks) by averaging all the measured bound fraction values. Mean ± SEM are plotted on the y axis as a bar chart. The promiscuity average was arbitrarily chosen as threshold value for the promiscuous group (S100A2, S100A4, S100P, S100A1, S100A6, S100A5, S100B, S100A10), while the rest of the S100ome is less promiscuous exhibiting fewer binding events.

The determined promiscuity parameter represents the binding properties of each S100 family member against the applied foldamer library (Fig. 4). Higher values (e.g. in the case of S100A2 or S100A6) implicate a promiscuous behavior with numerous fragments to interact with, while lower values belong to S100 members (e.g. S100A7 or S100A13) with only a few, weak interactions or without a clear binding preference.

## Discussion

### High-throughput holdup screening with foldamer libraries is a potent tool for specificity profiling of protein families

Using the H14 library, the chemical-binding preferences of the S100ome were screened effectively by the application of a HTP-HU assay, in which numerous strongly interacting foldamers were identified. When considering our overall results, the quantity and quality of the selected foldamer residues were utilized to create the specificity map of the overall S100ome. The detected enrichment of the highly hydrophobic and/or aromatic residues on the interacting surface is not a unique feature of the foldamers; moreover, their side chain binding propensities are biomimetic. Certain aromatic and aliphatic amino acids (i.e. Trp/Phe/Tyr and Leu/Ile/Val) were especially favored on the binding interface and these findings are in line with literature data from protein–protein interaction interface databases^13^. In general, selective recognition of ligands can be explained with the unique binding patterns of the protein interfaces; therefore, the side chain frequency levels can be different even for proteins having considerably similar structures. Importantly, as other foldamer libraries with different constitution (i.e. the constitutional and/or spatial conformation of the β^3^-amino acid side chains in the foldamer fragments) are available (e.g. the H12 foldamer library), the affinity of the individual S100 members towards the foldamer libraries can vary. Therefore, it would be interesting to screen the S100ome against other foldamer libraries, which could reveal additional relationships between the S100 members through their binding properties, providing a more refined specificity map of the family.

To validate the detected interactions, an orthogonal biophysical method, direct FP technique was used. Importantly, the affinity profile of the S100ome against the selected foldamers shows good correlation with the specificity map of the S100ome from our previous work using natural S100 partners^7^. While S100 proteins with multiple natural interaction partners (e.g. S100B, S100A6) are keen on binding foldamers, S100 proteins without a clear binding preference (S100G, S100A13) can barely interact with foldamer fragments, either because they only bind to proteins or peptides presenting a different conformation, or because they do not naturally bind to proteins.

Importantly, as S100 proteins are potential therapeutic targets, therefore the concatenation of the smaller foldamer fragments screened here by the HTP HU assay might lead to highly specific and strong ligands, paving the way to rational drug design.

### The promiscuity of the full S100ome is explained using the foldamer library

Promiscuity (or its complementary notion, specificity) within a protein family can hardly be defined against natural partners, owing to still potentially unknown interactions. However, using the foldamer library against the S100ome to screen the binding properties within the protein family, promiscuity can be defined for each member against the actual library, which eventually may approximate the real, yet undefined promiscuity profile. The promiscuity parameter values defined in this study for each S100 member against the foldamer library are in good correlation with previous works^7,18,24–30^. Promiscuous S100 proteins with several known cellular partners (e.g. S100A6 or S100A4) show more interactions towards the members of the foldamer library, thus displaying a higher value of their promiscuity parameter. Orphan S100 proteins without a clear intra- or extracellular binding preference (e.g. S100A16 or S100Z) exhibit less interactions with lower binding affinity, which is represented by a lower value of the promiscuity parameter. Of note, we excluded few amino acids, due to the lack of any proteogenic side chain (G), the potential disruptive effect on the helical structure (P), uncontrolled disulfide formation (C), difficulties associated with the monomer synthesis (H)^31^. Nevertheless, the 16 proteogenic side chains in our library can screen the binding properties of the S100ome without leading to a significant cavity.

Orphan members do not interact considerably with the members of the H14 foldamer library, thus suggesting that these S100 proteins might lack the ability to interact with proteins in the real cellular environment and rather play a role in the Ca^2+^-homeostasis^32^. It is still possible that the less promiscuous S100 proteins have highly specific, yet undiscovered natural interaction partners that may adopt a drastically different, non-helical conformation, explaining their lack of preference for the H14 helical foldamer fragments.

While, in principle, functional redundancy within the S100ome can only be interpreted with natural partners, screening the S100ome against ‘non-natural’ libraries constitutes a powerful approach to draw a more detailed and refined picture about binding properties within the family. The promiscuity of the S100 proteins observed herein against the foldamer library, may have high relevance for their actual interactome in the real cellular environment.

## Methods

### S100 protein expression and purification

S100 proteins (UniProt accession codes: S100A1: P23297, S100A2: P29034, S100A3: P33764, S100A4: P26447, S100A5: P33763, S100A6: P06703, S100A7: P31151, S100A8: P05109, S100A9: P06702, S100A10: P60903, S100A11: P31949, S100A12: P80511, S100A13: Q99584, S100A14: Q9HCY8, S100A15: Q86SG5, S100A16: Q96FQ6, S100B: P04271, S100G: P29377, S100P: P25815 and S100Z: Q8WXG8) were expressed and purified with N-terminal His_6_-tag as described previously^33^. Briefly, S100 proteins were cloned into a modified pET15b vector with a TEV protease cleavable N-terminal His_6_-tag and expressed in *Escherichia* coli BL21 (DE3) cells, followed by Ni^2+^-affinity chromatography. For HU assay, S100 proteins were further purified by either hydrophobic interaction chromatography or ion exchange chromatography without the cleavage of the N-terminal His_6_-tag applying standard conditions^33^. For direct FP measurements, the N-terminal His_6_-tag was cleaved, and the S100 proteins were purified by hydrophobic interaction chromatography, ion exchange chromatography or size exclusion chromatography^33^. The quality of the recombinant proteins was checked by SDS-PAGE analysis in all cases. The concentration of the recombinant S100 proteins was determined by UV spectrophotometry using the absorbance of Tyr and Trp residues.

### Synthesis and purification of the foldamer libraries

The foldamer libraries were synthetized and purified as described previously^34^. Briefly, the 256-memberd library was divided to four sublibraries (aromatic, charged, apolar, non-charged polar) containing 64 members (Fig. S13). The libraries were synthetized with a CEM liberty 1 microwave peptide synthesizer using HATU (1-[bis(dimethylamino)methylene]-1H-1,2,3-triazolo[4,5-b]-pyridinium-3-oxid hexafluorophosphate) as coupling agent following Fmoc strategy by coupling aminocyclohexanecarboxylic acids and β^3^-amino acids. After cleavage of the sublibraries, the samples were lyophilized and the mixtures of foldamers were purified by RP-HPLC (Phenomenex Luna C18, 250 × 10 mm), followed by HPLC–MS identification. The purity and equimolarity of the foldamer libraries were checked by HPLC–MS.

### Synthesis and purification of labeled foldamer sequences

Individual foldamers were synthetized manually using solid-phase peptide synthesis with Fmoc strategy applying HATU as coupling agent^15^. Coupling of the 5(6)-carboxyfluorescein to the ε-amino group of a Lys attached to the C-terminus of the foldamers was carried out as the last step of the synthesis. The crude foldamers were cleaved from the resin and then, the samples were precipitated in diethyl ether and purified by RP-HPLC (Phenomenex Jupiter C18, 250 × 10 mm). Purity was confirmed by HPLC–MS. The concentration of the foldamers was determined by UV-spectrophotometry using the absorbance of 5(6)-carboxyfluorescein.

### Synthesis and purification of the peptide TRTK12

The TRTK12 peptide was synthetized as described previously^7^. Briefly, the peptide was chemically synthetized by solid phase peptide synthesis with a PS3 peptide synthesizer (Protein technologies, Tucson, AZ, USA) using Fmoc/tBu strategy, and purified by RP-HPLC using a Jupiter 300 Å C_18_ column.

### Investigation of solubility by light scattering

Solubility of the foldameric fragments is presented through the highly hydrophobic WW and its carboxyfluorescein derivative fWW. Foldamers were dissolved in a buffer consisting of 20 mM HEPES, 150 mM NaCl, 2 mM CaCl2, pH = 7.5 at 11 different concentrations from 10 nM to 1 mM. 100 μl samples in triplicates were pipetted into a 96-well ELISA plate and absorbance values were recorded by using FLUOstar OPTIMA plate reader at 650 nm wavelength.

### Holdup assay

Screening the interaction between the foldamer libraries and the S100ome was performed by holdup assays as described previously^34^. Briefly, S100 proteins were immobilized in a buffer containing 20 mM HEPES pH 7.5, 150 mM NaCl, 2 mM CaCl_2_, 1 mM TCEP on Co^2+^-affinity resin (~ 2 mg protein / ml resin concentration) via the N-terminal His_6_-tag followed by the addition of the foldamer libraries. After incubation, the resin was centrifuged (*Pierce™ Spin Cups—paper filter, Thermo Fisher Scientific*) to separate the unbound fraction of the library. Negative controls were prepared using the procedure described above in the absence of the His_6_-tagged protein. The flow-through fractions were analyzed by HPLC–MS (Fig. S17). Quantitative evaluation of the HPLC–MS chromatograms were performed with Thermo Xcalibur software. Bound fractions (F_B_) were calculated by the following equation (Eq. 2) from the loss of intensity of the foldamer fragments (AUC_protein_) in the flow-through fractions compared to the control samples (AUC_control_).

2 $${F}_{B}=1- \frac{{AUC}_{protein}}{{AUC}_{control}}$$

### Calculation of amino acid preference

For each 16 amino acid, a summarized F_B_ (F_B_^aa^) was calculated by the following equation in the instances of all S100 proteins:

3 $$\overline{{F }_{B}^{aa}}=\sum {F}_{B}^{aa, 2p}+ \sum {F}_{B}^{aa,5p}$$

In which F_B_^aa,2p^ and F_B_^aa,5p^ are fraction bound values of foldamer fragments containing the proteogenic sidechain of interest in the 2nd or 5th position, respectively. The amino acids were further categorized into five groups (aromatic: F, W, Y; aliphatic: A, I, L, M, V; polar: N, Q, S, T; acidic: D, E; basic: K, R), and the root fraction bound values (F_B_^root^) were calculated for each group in the case of all S100 proteins by the following equation:

4 $$\overline{{F }_{B}^{root}}= \sum_{m=1}^{K}\overline{{{F }_{B}^{aa}}_{m}}$$

In which K is the number of amino acids in the individual groups. Standard deviation and standard error were calculated through propagation of uncertainty using the standard formula^35^.

### Fluorescence polarization assay

In direct fluorescence polarization assays, S100 proteins were diluted in a buffer containing 50 nM labeled foldamer, 20 mM HEPES pH 7.5, 150 mM NaCl, 1 mM CaCl_2_, 0.5 mM TCEP and 0.01% Tween20. The dilution series (50 µl) were divided into three technical repeats and transferred (15 µl) to a 384-well microplate. In competitive fluorescence polarization assays, the buffer applied in direct measurements was supplemented with the S100 protein of interest to reach a saturation of 60–80%. This mixture was titrated with the competitor (i.e. the unlabeled peptide). Fluorescence polarization was measured in 8 different S100 concentrations (one of which contained no S100 protein) on a Synergy H4 plate reader using 485 ± 20 nm and 528 ± 20 nm band-pass filters for excitation and emission, respectively. The K_d_ values were obtained by fitting the data from the FP measurements with the python-based ProFit software using quadratic and competitive binding equation for direct and competitive FP, respectively^7^. The detection threshold was based on two parameters. First, we rejected all fitted dissociation constants above 1 mM. Second, we also rejected all fitted data where the experimental window was significantly lower (< 80 mP) or higher (> 350 mP), compared to other, stronger interactions of the same labeled foldamer.

### Correlation between holdup and FP

The correlation between the holdup assay (F_B_ values) and the fluorescence polarization (K_d_ values) was quantitatively described by the Pearson correlation coefficient (PCC) using the standard formula.

### Calculation of promiscuity

Promiscuity, defined as a number between 0 (no interaction with any member) and 1 (the strongest interaction with all the members in the library) was calculated for each S100 protein according to Eq. (1) by averaging the measured F_B_ values.

## Supplementary Information

Supplementary Information 1.

Supplementary Information 2.
