# Supplementary material for: Promiscuity mapping of the S100 protein family using a high-throughput holdup assay
Source: Sci Rep. 2022 Apr 7;12:5904. doi: 10.1038/s41598-022-09574-2 (PMC8991199; doi:10.1038/s41598-022-09574-2)
Supplement: Supplementary file 1 — Supplementary Information 1. [file 41598_2022_9574_MOESM1_ESM.pdf]

Promiscuity mapping of the S100 protein family using a high-throughput holdup assay

Márton A. Simon[1, 2, $], Éva Bartus[3, 4 $], Beáta Mag [3], Eszter Boros[1], Lea Roszjár[3], Gergő Gógl[1, 5], Gilles Travé[5], Tamás A. Martinek[3, *] and László Nyitray[1, *]

[1]Department of Biochemistry, Eötvös Loránd University, H-1117 Budapest, Hungary

[2]Department of Medical Biochemistry, Semmelweis University, H-1094 Budapest, Hungary

[3]Department of Medical Chemistry, University of Szeged, H-6720 Szeged, Hungary

[4]MTA-SZTE Biomimetic Systems Research Group, University of Szeged, H-6720 Szeged, Hungary

[5]Equipe Labellisee Ligue 2015, Department of Integrated Structural Biology, Institut de Genetique et de Biologie Moleculaire et Cellulaire (IGBMC), INSERM U1258/CNRS UMR 7104/Universite de Strasbourg, 1 rue Laurent Fries, BP 10142, F-67404 Illkirch, France

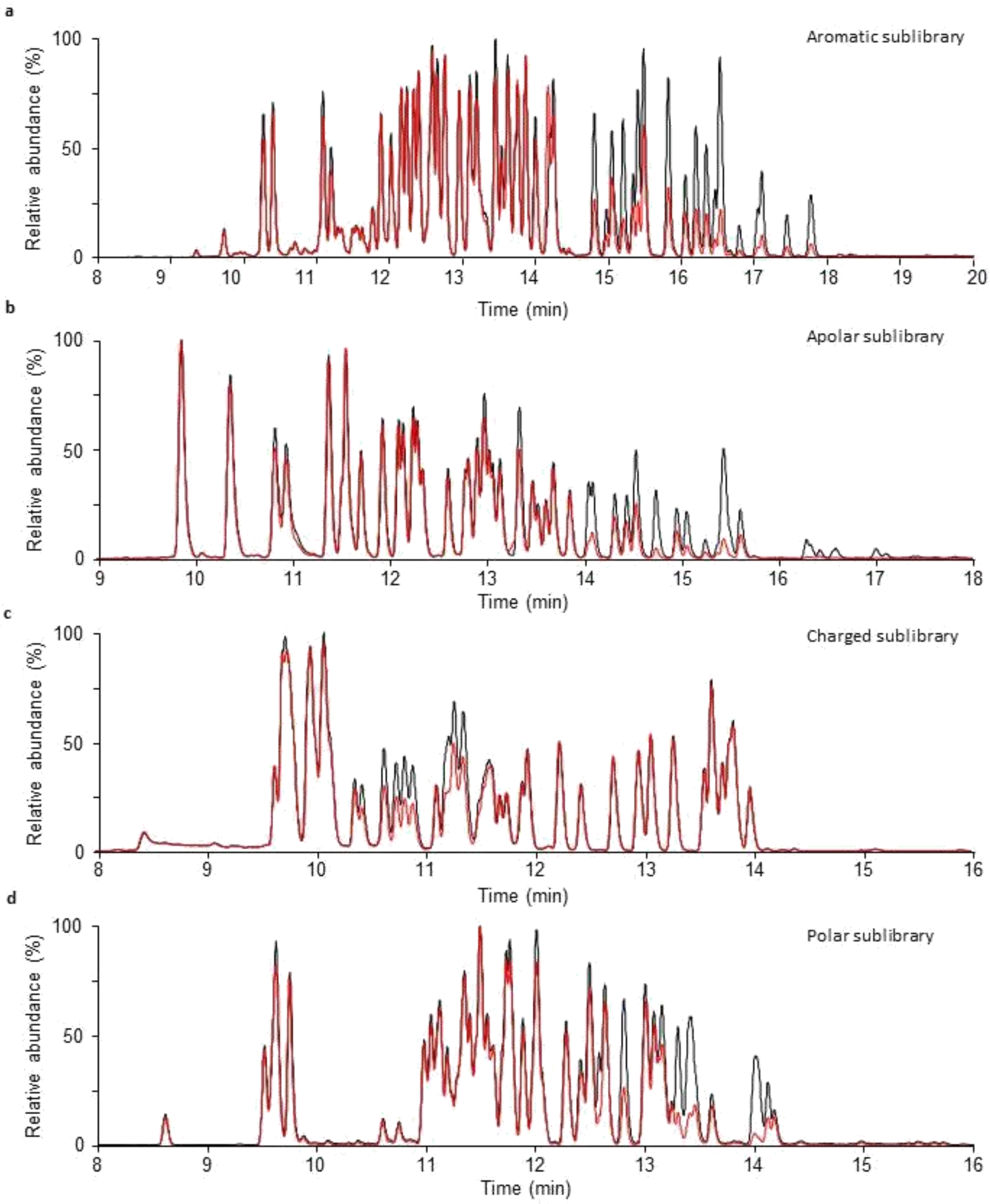

Figure S1. Representative HPLC-MS trace. Extracted ion chromatograms of aromatic (a), aliphatic (b), charged (c) and polar (d) sublibraries. Control samples represented with black color contain all library members, whereas red line shows the chromatogram of flow through fraction after the interaction with S100A4 protein. Samples were injected onto Phenomenex Aeris WIDEPORE XB-C18 column and the following gradient elution method was applied uniformly: 5% to 80% eluent B during 25 min with flow rate of 0.7 mL min-1.

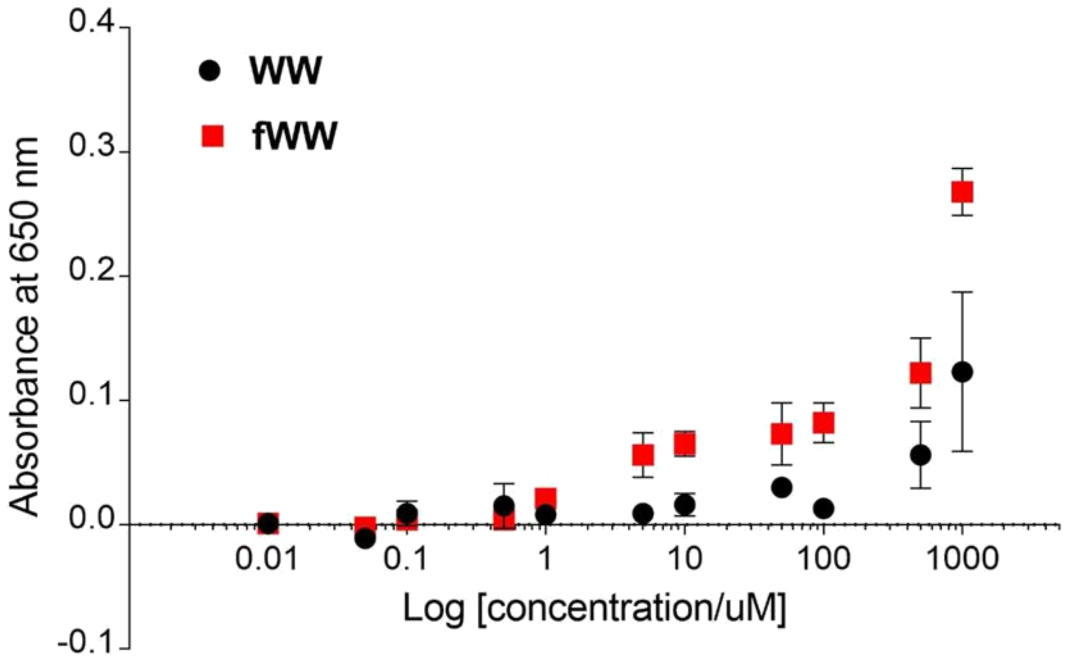

Figure S2. Investigation of solubility of the labeled and unlabeled version of a highly hydrophobic foldamer fragment by light scattering. Different concentrations of WW (black circle) and fWW (red square) were examined by absorbance measurement at 650 nm (See methods) and plotted as mean + SEM The unlabeled WW fragment possesses increased light scattering above 500 µM, while the labeled fragment exerts significantly higher light scattering above 1 µM.

Interaction between the S100ome and the LSM library measured with holdup assay

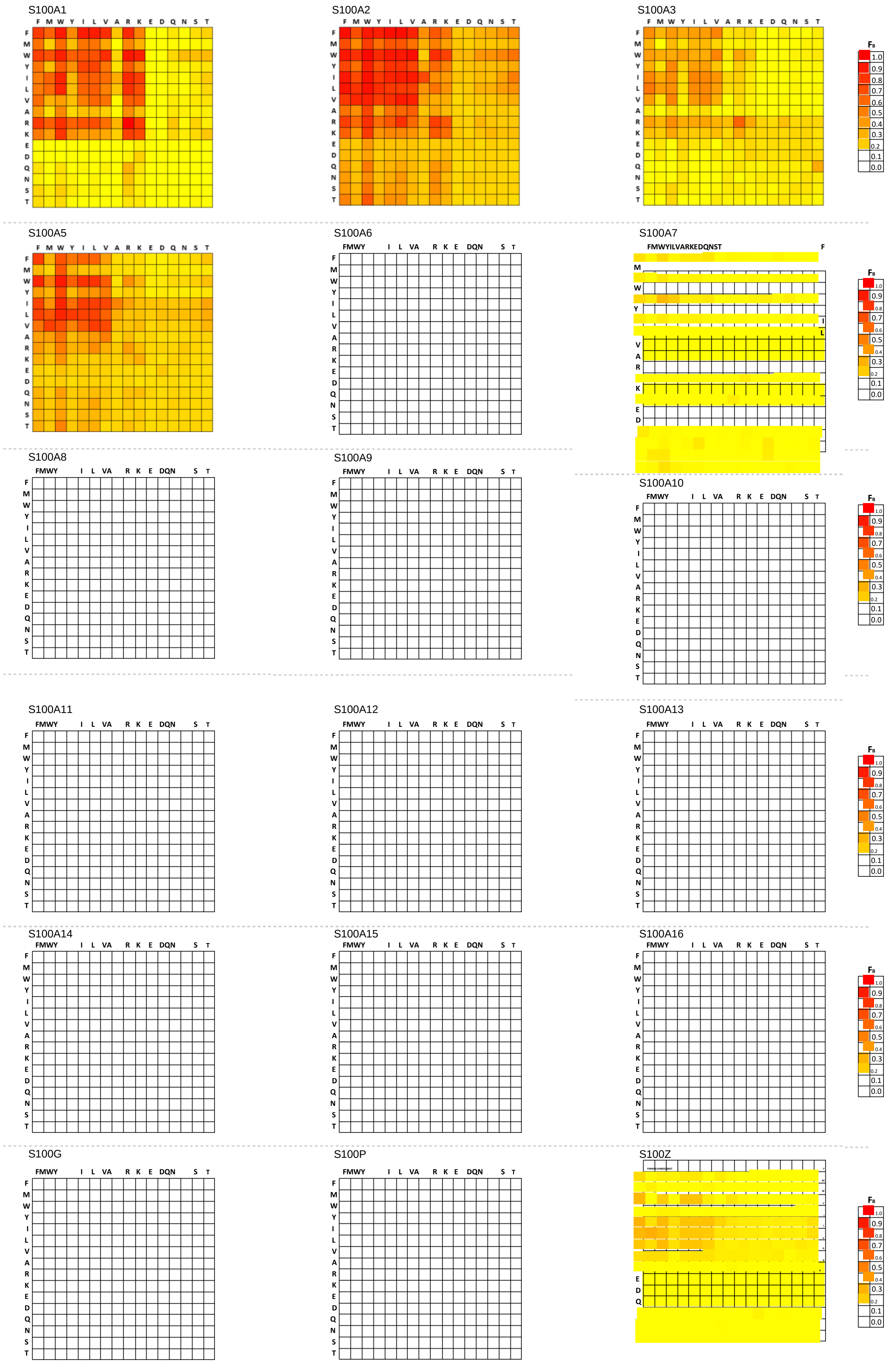

Figure S3. The binding affinities of the S100ome towards the H14 foldamer library measured by a high-throughput holdup assay. The calculated F_B values were depicted as heat maps on a linear scale. F_B ranges are color-coded as shown on the right. The x and y axis represent the amino acids in the 5th and 2nd positions, respectively. The missing S100 proteins can be found in following reference [14].

S100ome – fIF measured by FP

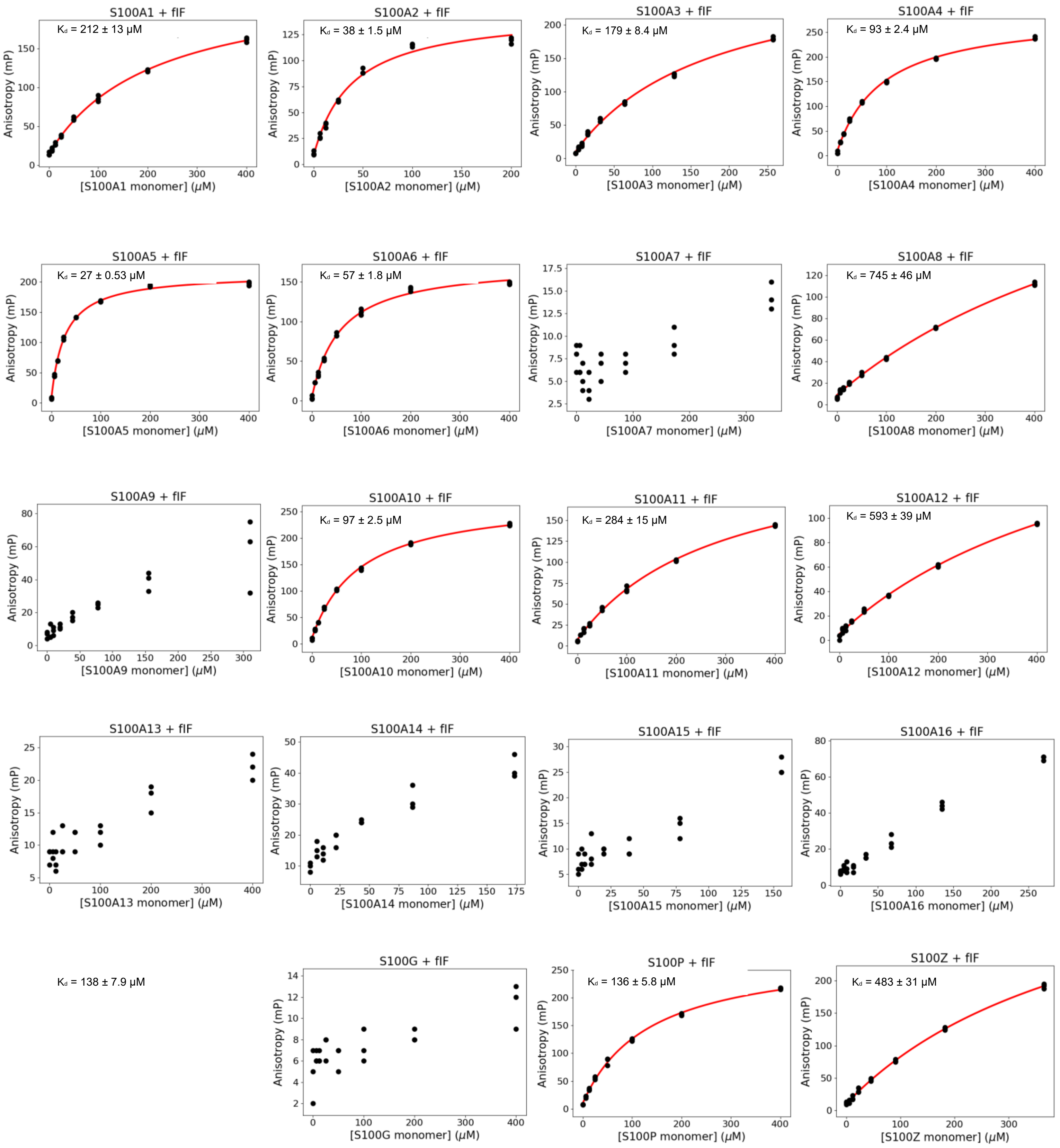

Figure S4. The interactions between the S100ome and fIF as measured by FP. Dissociation constants (mean + SEM) were calculated by fitting the anisotropy values (mP) using quadratic equation with the ProFit program.

S100ome – fIL measured by FP

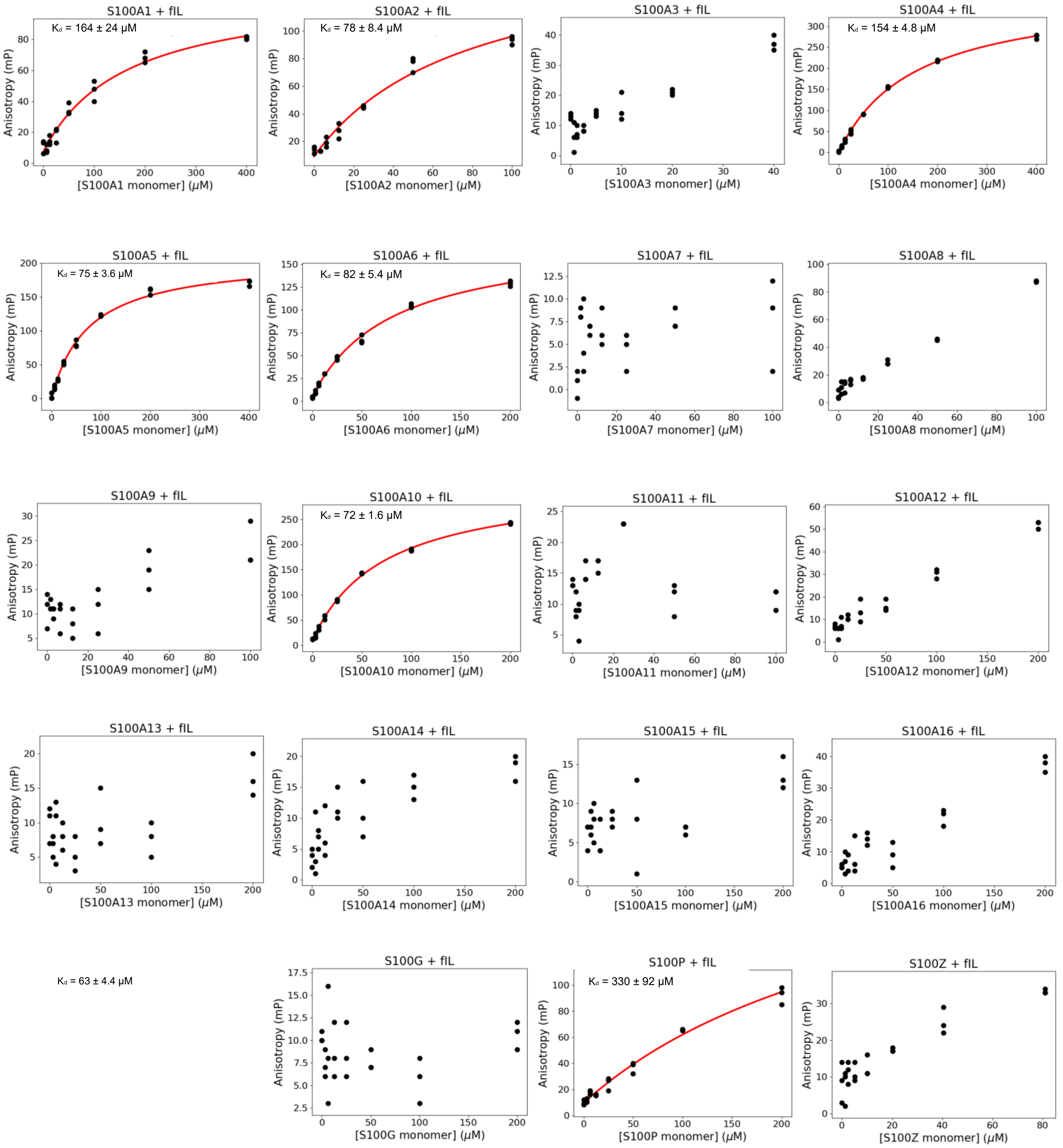

Figure S5. The interactions between the S100ome and fIL as measured by FP. Dissociation constants (mean + SEM) were calculated by fitting the anisotropy values (mP) using quadratic equation with the ProFit program.

S100ome – fRF measured by FP

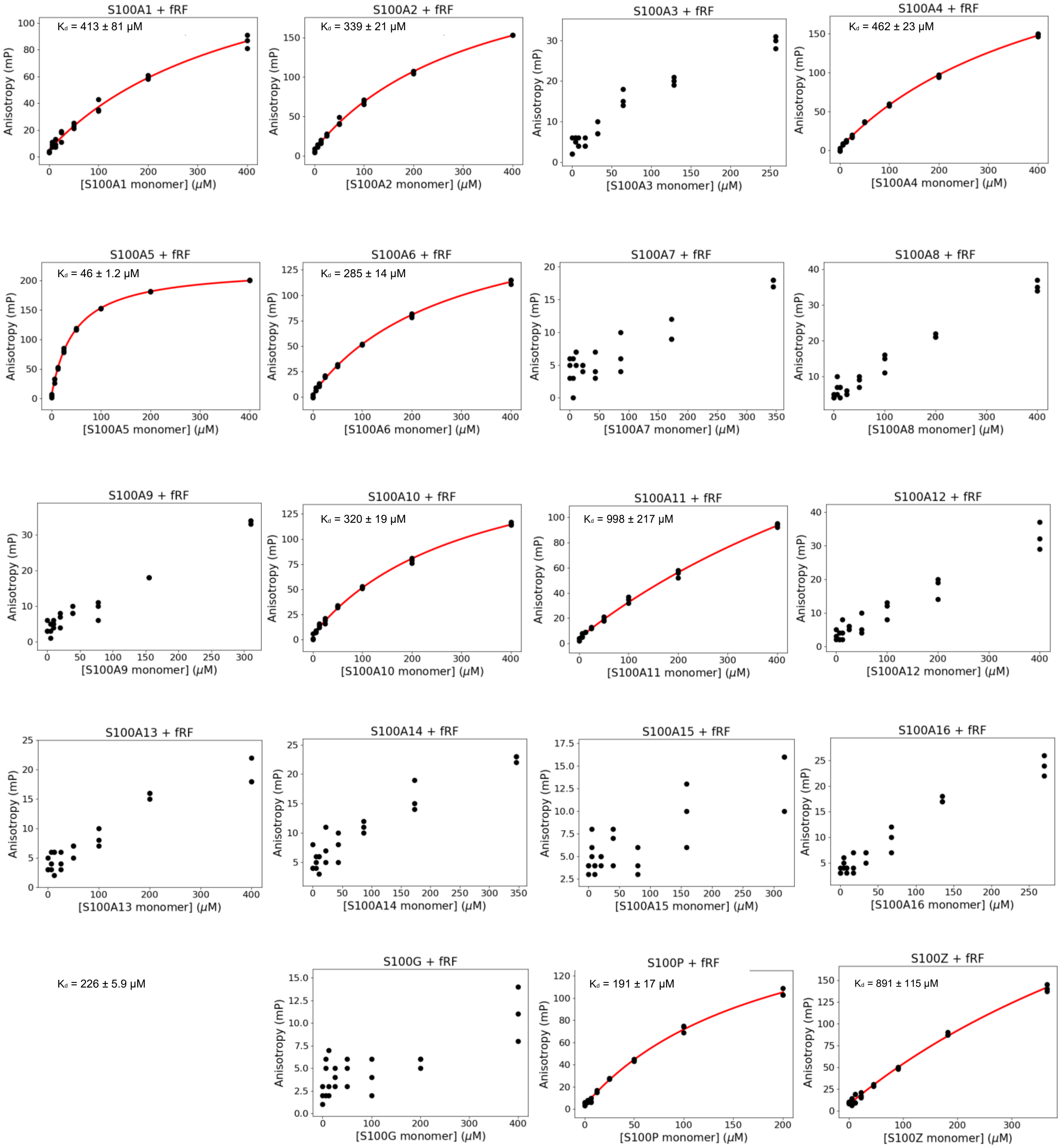

Figure S6. The interactions between the S100ome and fRF as measured by FP. Dissociation constants (mean + SEM) were calculated by fitting the anisotropy values (mP) using quadratic equation with the ProFit program.

S100ome – fRR measured by FP

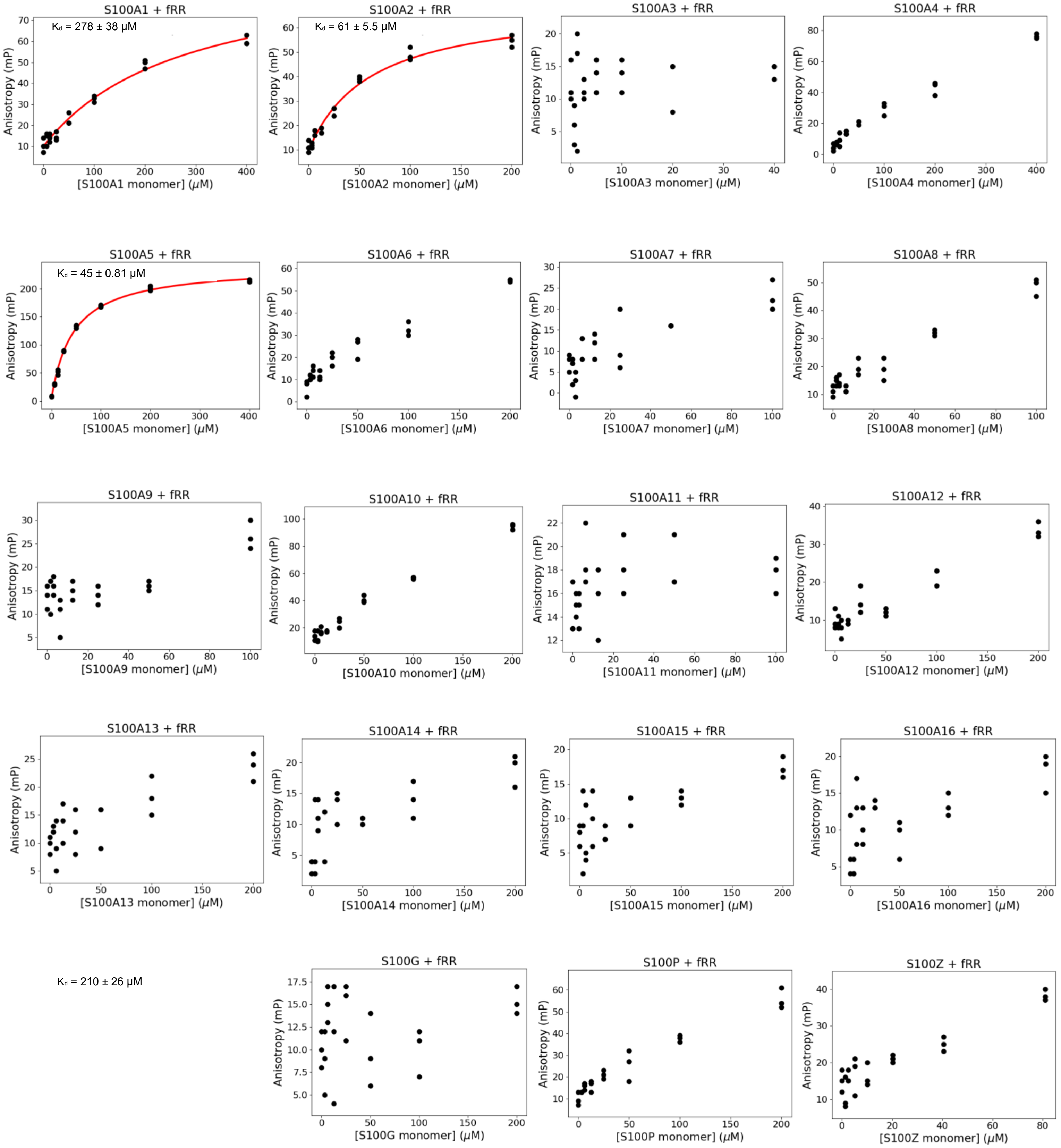

Figure S7. The interactions between the S100ome and fRR as measured by FP. Dissociation constants (mean + SEM) were calculated by fitting the anisotropy values (mP) using quadratic equation with the ProFit program.

S100ome – fTI measured by FP

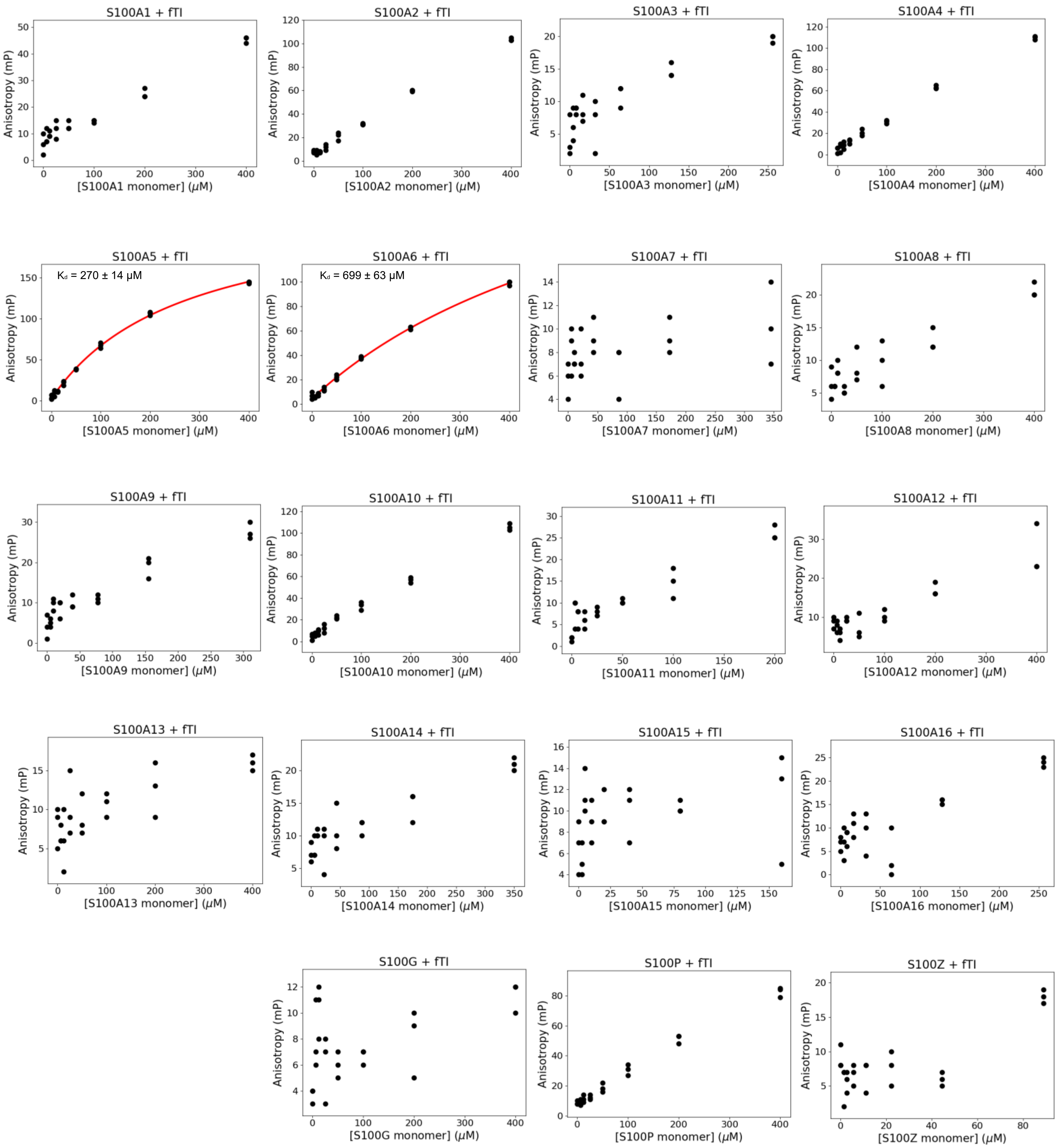

Figure S8. The interactions between the S100ome and fTI as measured by FP. Dissociation constants (mean + SEM) were calculated by fitting the anisotropy values (mP) using quadratic equation with the ProFit program.

S100ome – fTM measured by FP

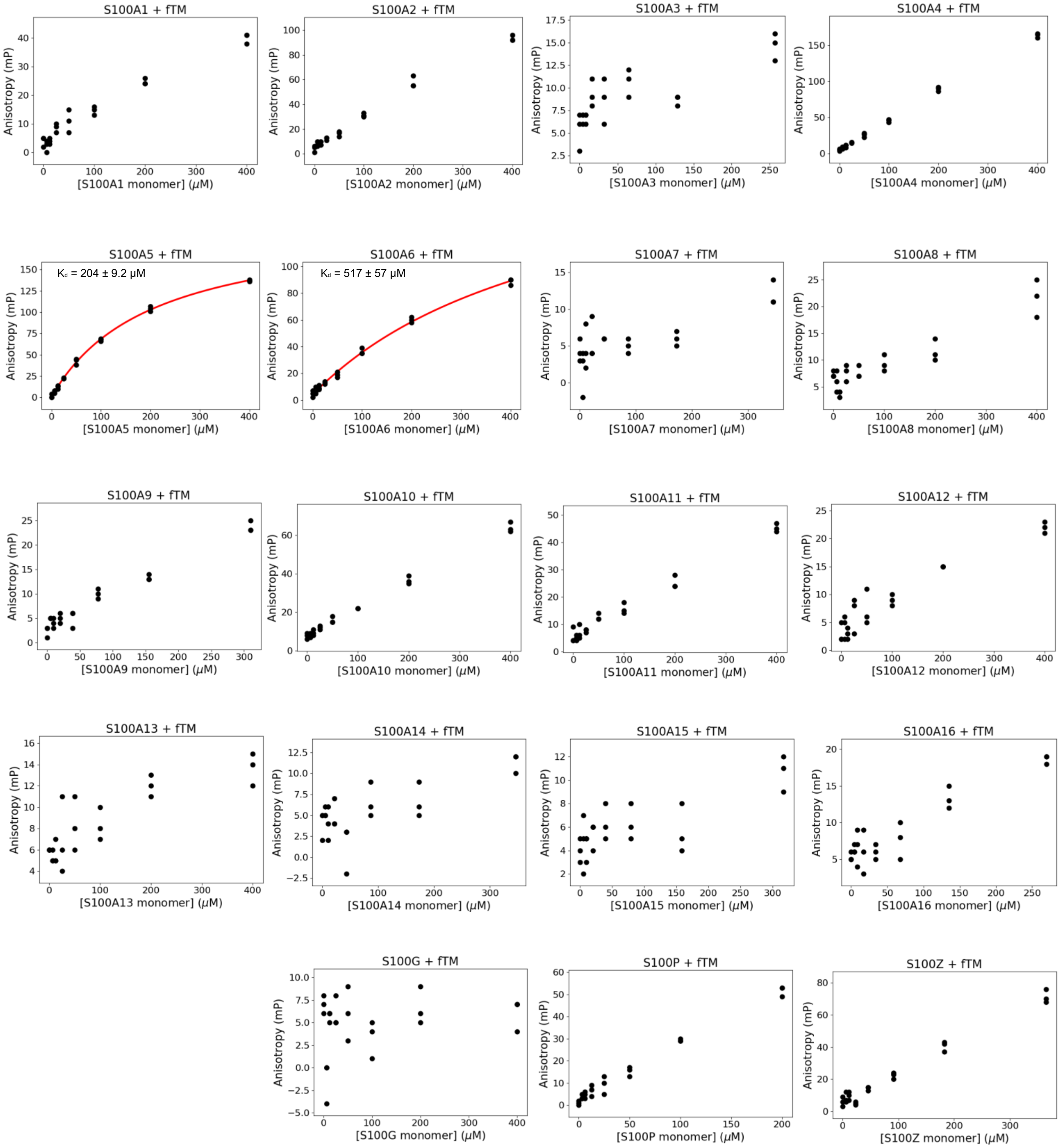

Figure S9. The interactions between the S100ome and fTM as measured by FP. Dissociation constants (mean + SEM) were calculated by fitting the anisotropy values (mP) using quadratic equation with the ProFit program.

S100ome – fTW measured by FP

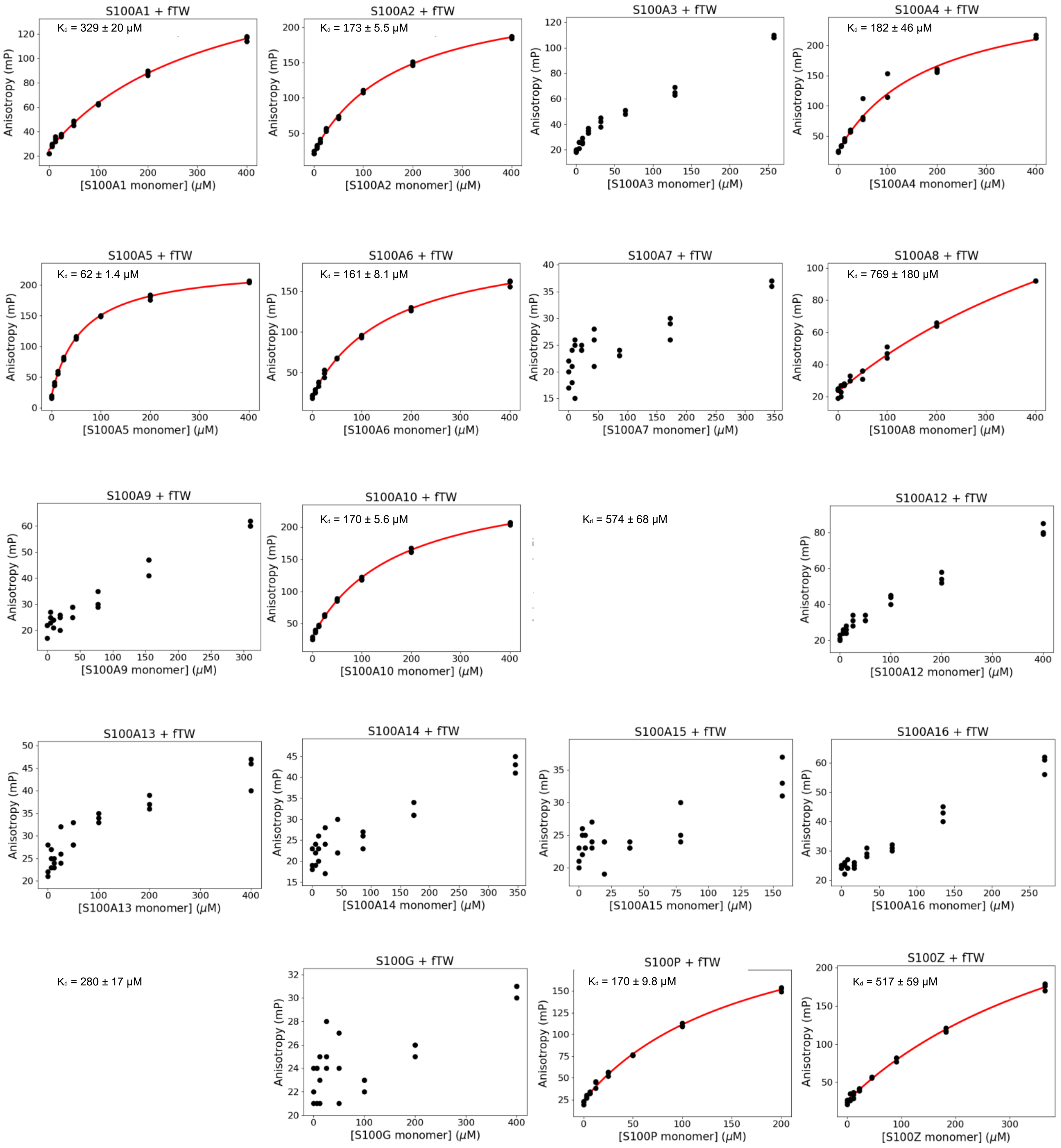

Figure S10. The interactions between the S100ome and fTW as measured by FP. Dissociation constants (mean + SEM) were calculated by fitting the anisotropy values (mP) using quadratic equation with the ProFit program.

S100ome – fVL measured by FP

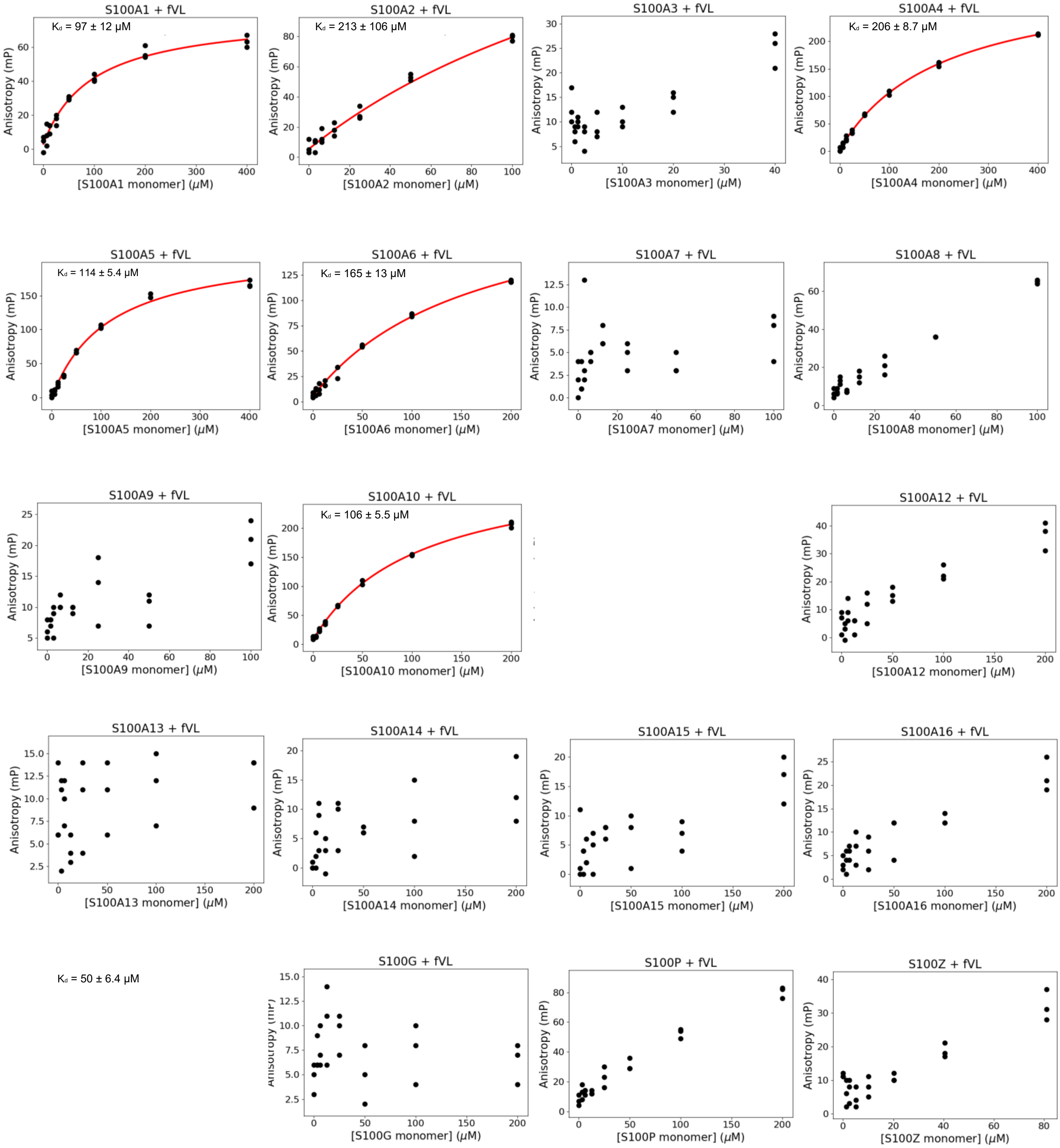

Figure S11. The interactions between the S100ome and fVL as measured by FP. Dissociation constants (mean + SEM) were calculated by fitting the anisotropy values (mP) using quadratic equation with the ProFit program.

S100ome – fWL measured by FP

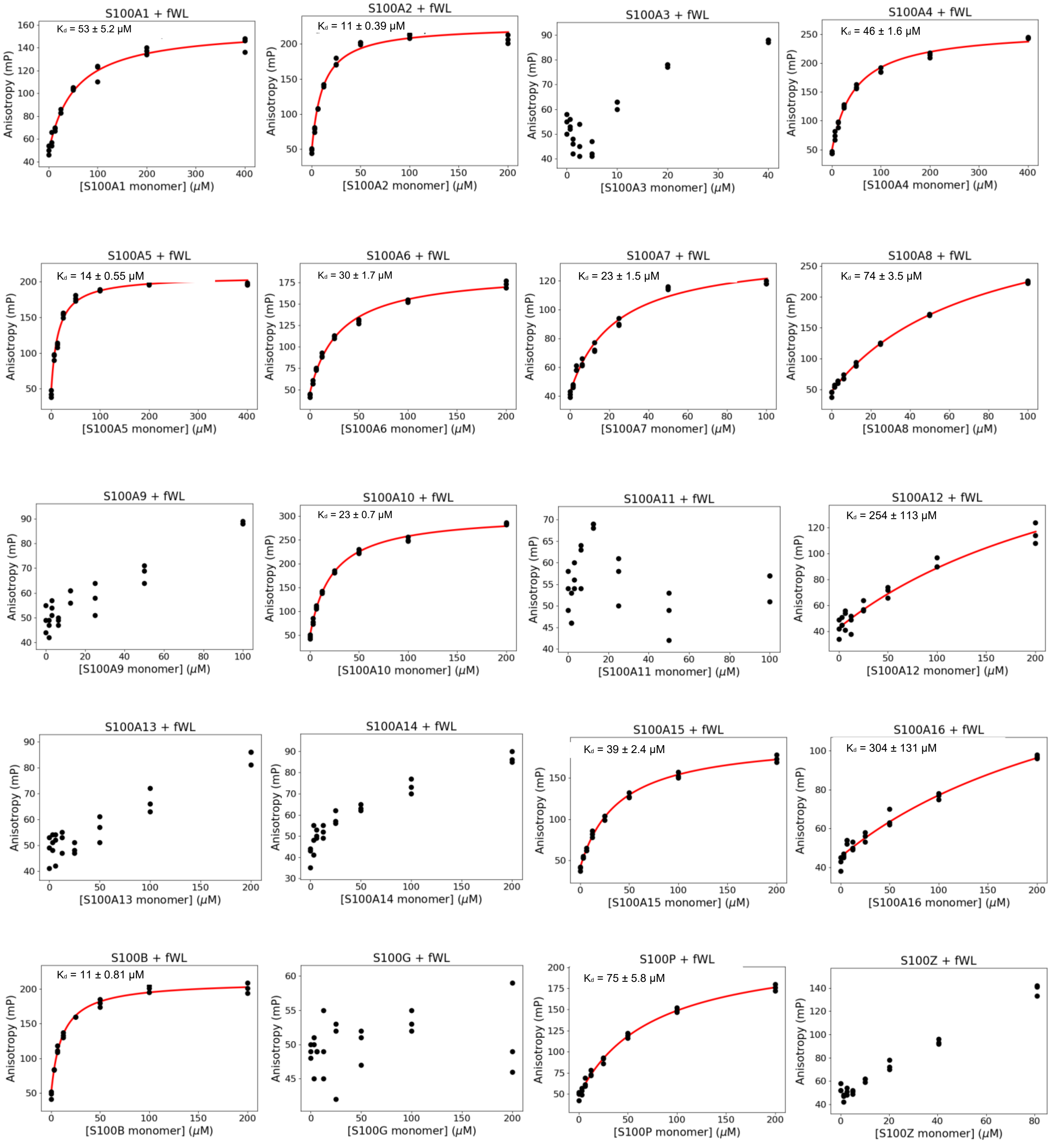

Figure S12. The interactions between the S100ome and fWL as measured by FP. Dissociation constants (mean + SEM) were calculated by fitting the anisotropy values (mP) using quadratic equation with the ProFit program.

S100ome – fWW measured by FP

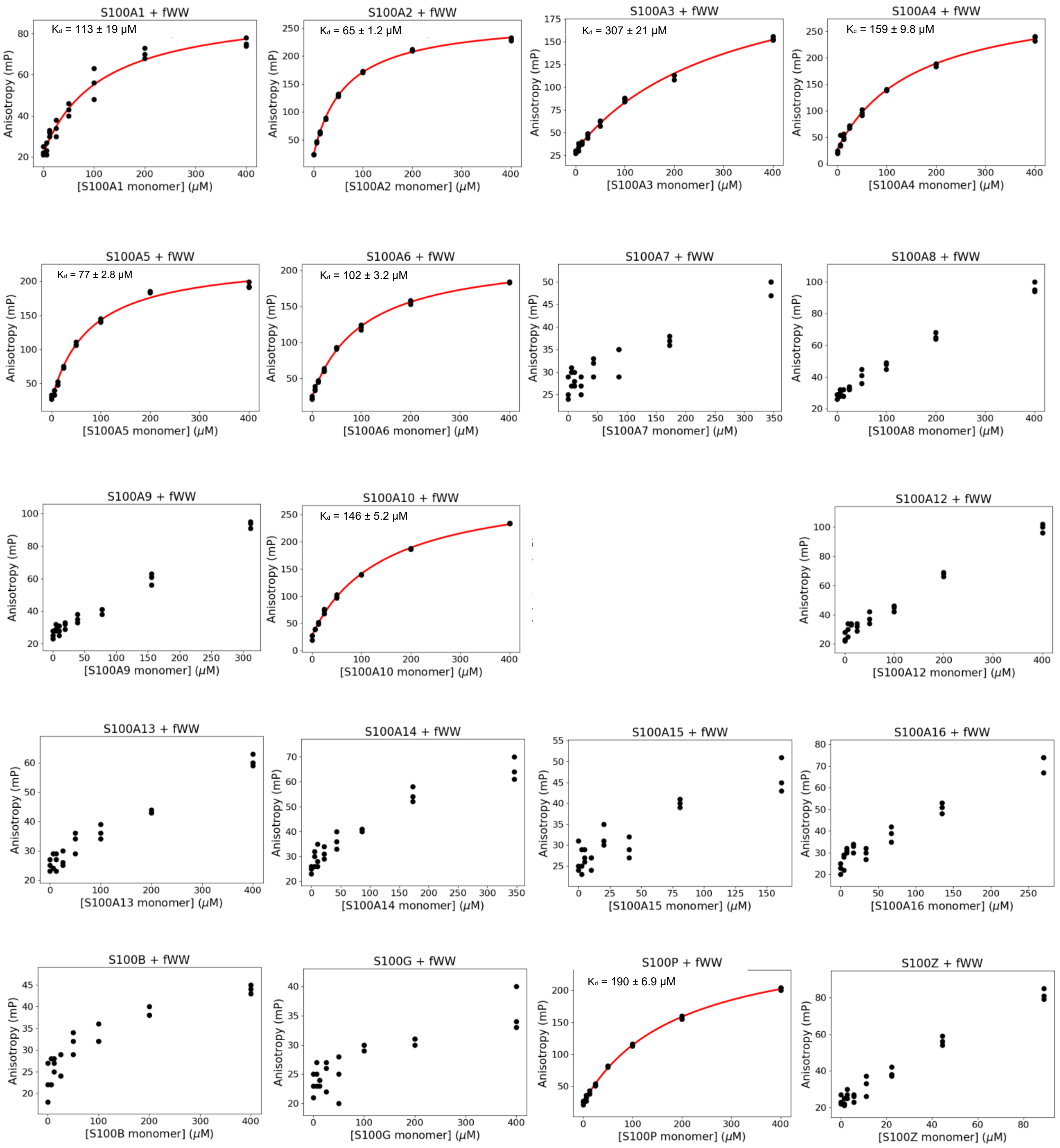

Figure S13. The interactions between the S100ome and fWW as measured by FP. Dissociation constants (mean + SEM) were calculated by fitting the anisotropy values (mP) using quadratic equation with the ProFit program.

S100ome – fYF measured by FP

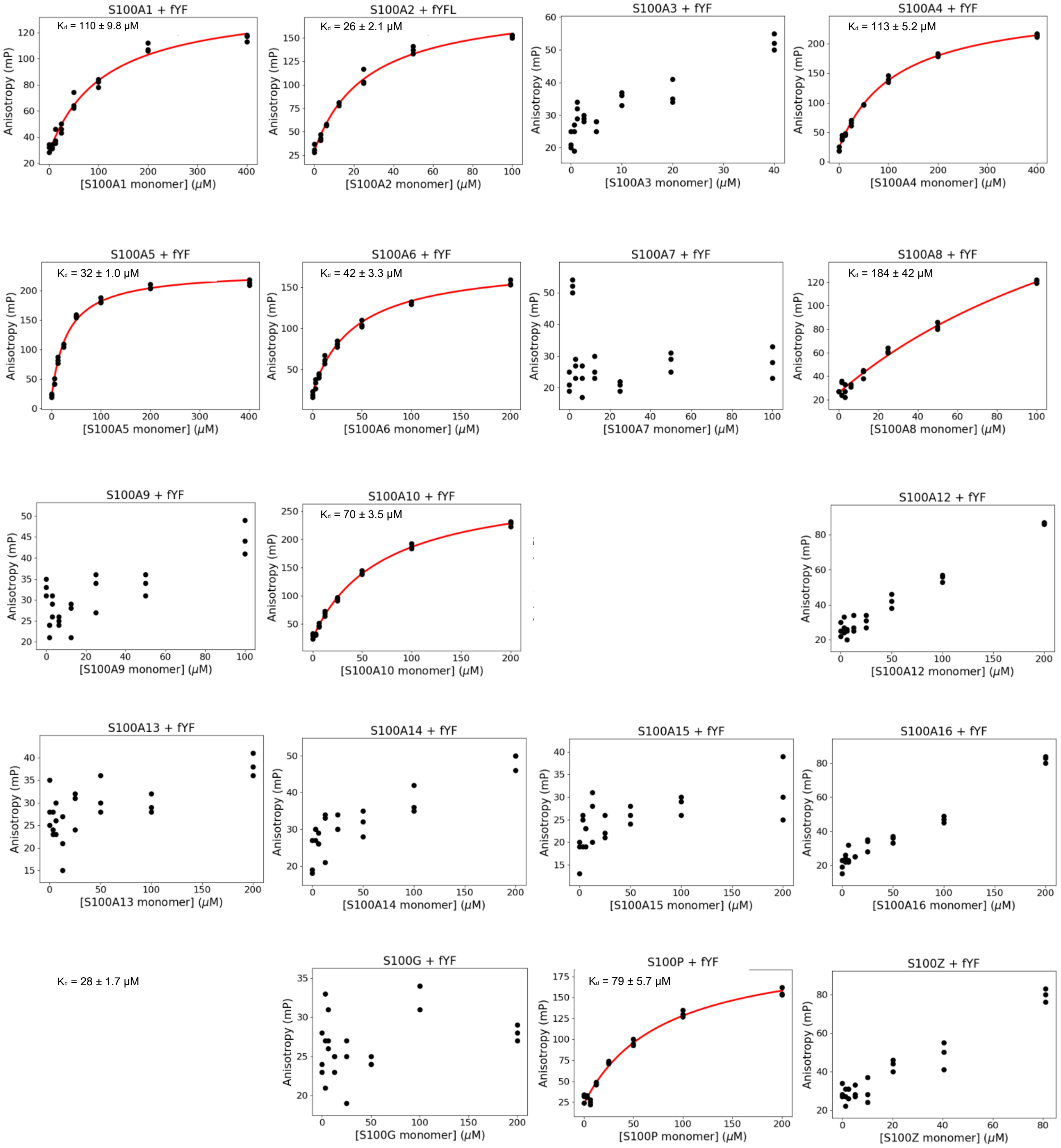

Figure S14. The interactions between the S100ome and fYF as measured by FP. Dissociation constants (mean + SEM) were calculated by fitting the anisotropy values (mP) using quadratic equation with the ProFit program.

The constructed labeled foldamers for FP

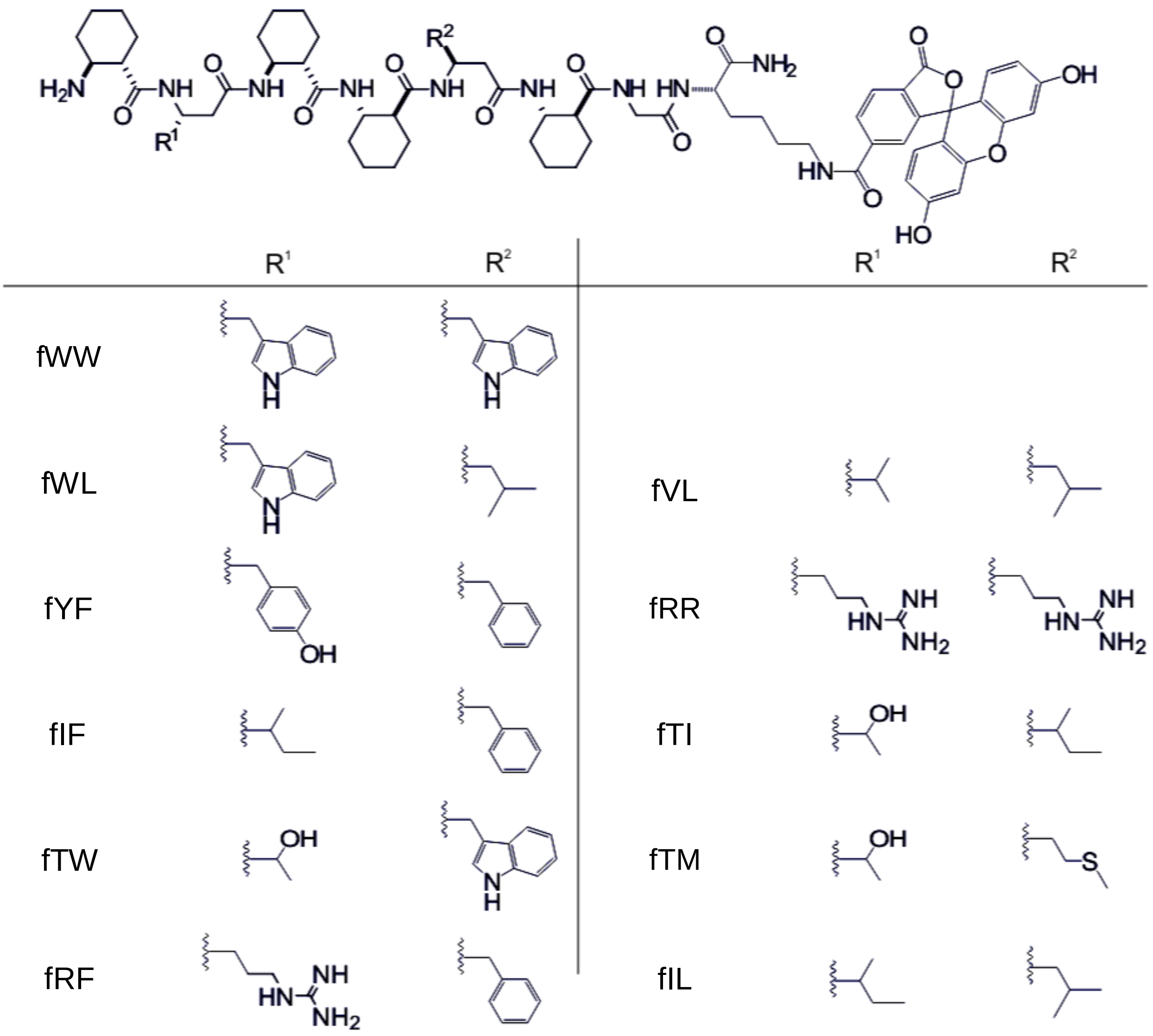

Figure S15. The structures of the selected foldamer sequences labeled with 5(6)-carboxyfluorescein. Foldamers were coupled to the fluorescence dye through two glycine residues.

MS Analysis of the chemically synthesized labeled foldamers for FP

Compound	Calculated MW (average)	Expected m/z values		Observed m/z vales	
		$[M+H]^+$	$[M+2H]^{2+}$	$[M+H]^+$	$[M+2H]^{2+}$
**fWW**	1461.06	1462.06	731.53	1462.57	731.61
**fWL**	1388.07	1389.07	695.04	1388.66	695.40
**fYF**	1399.04	1400.04	700.52	1399.44	700.62
**fIF**	1349.06	1350.06	675.53	1349.94	676.05
**fTW**	1376.03	1377.03	689.02	1377.12	689.63
**fRF**	1392.08	1393.08	697.04	1394.06	697.42
**fII**	1315.08	1316.08	658.54	1315.73	658.77
**fVL**	1301.06	1302.06	651.53	1301.62	651.89
**fRR**	1401.12	1402.12	701.56	1402.75	701.76
**fTI**	1303.04	1304.04	652.52	1303.77	652.75
**fTM**	1321.00	1322.00	661.50	1321.81	661.76
**fIL**	1315.08	1316.08	658.54	1315.75	658.81

Table S1. Summary of the ESI-MS data for the selected foldamers labeled with the fluorescence dye.

LC-MS Analysis of the chemically synthesized labeled foldamers for FP

Compound: **fWW**

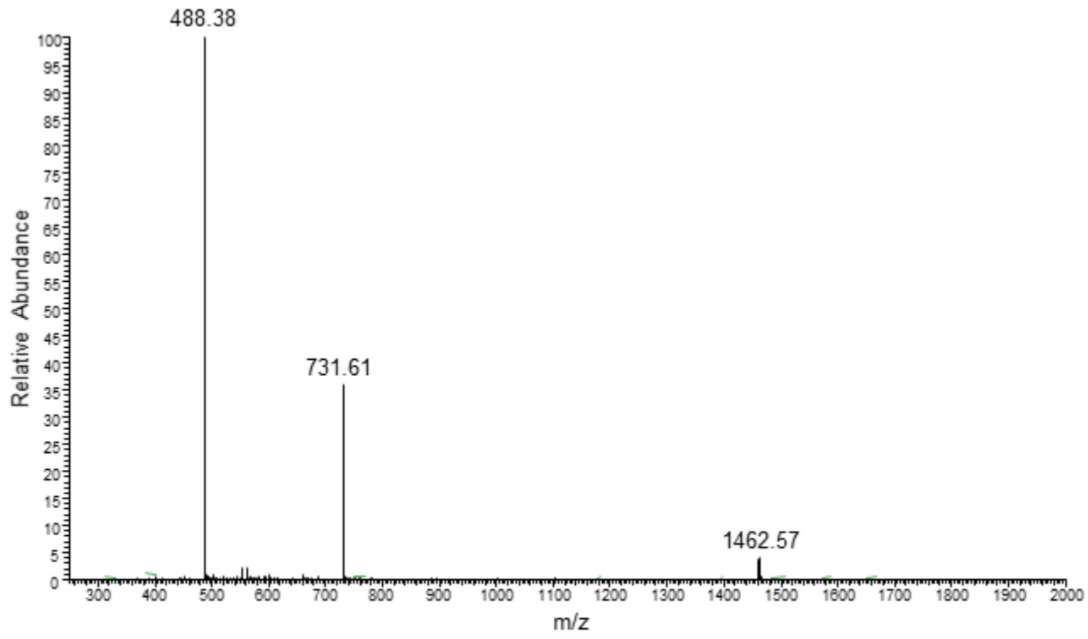

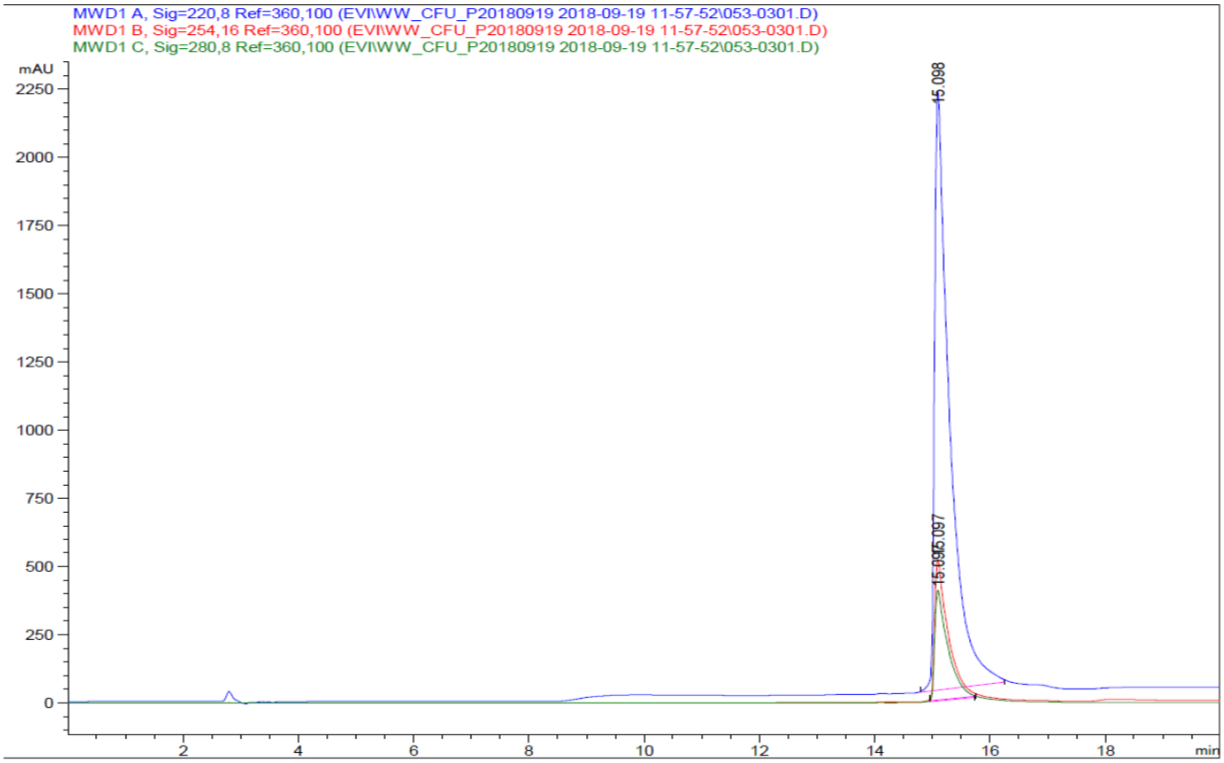

Figure S16. The MS spectra and the HPLC chromatogram of fWW (Column: Phenomenex Luna C18 (250 x 4.6 mm, particle size: 5 micron, pore size: 100 Å); Gradient: 5-80% 20min 1.2 mL min^{-1}).

Compound: **fWL**

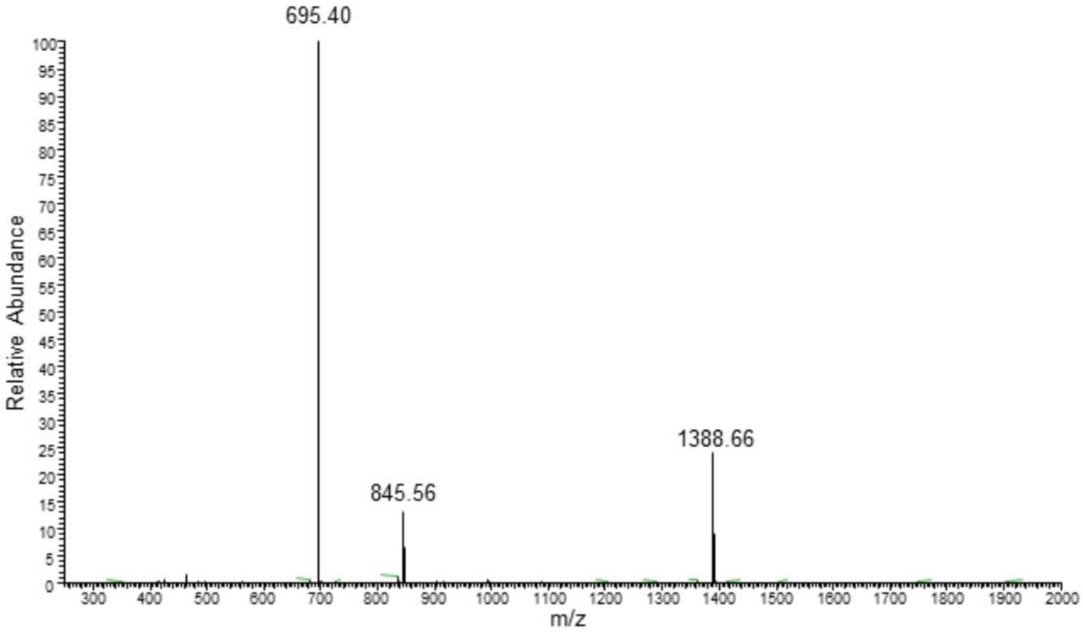

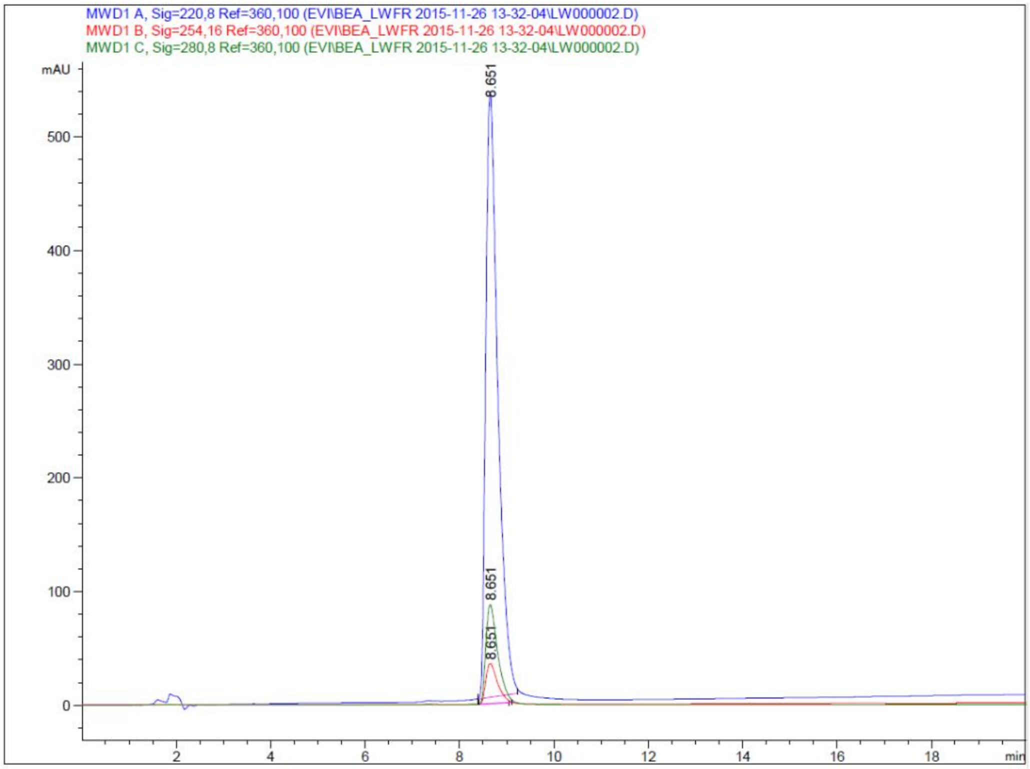

Figure S17. The MS spectra and the HPLC chromatogram of fWL (Column: Phenomenex Luna C18 (250 x 4.6 mm, particle size: 5 micron, pore size: 100 Å); Gradient: 60-80% 20min 1.2 mL min^{-1}).

Compound: **fYF**

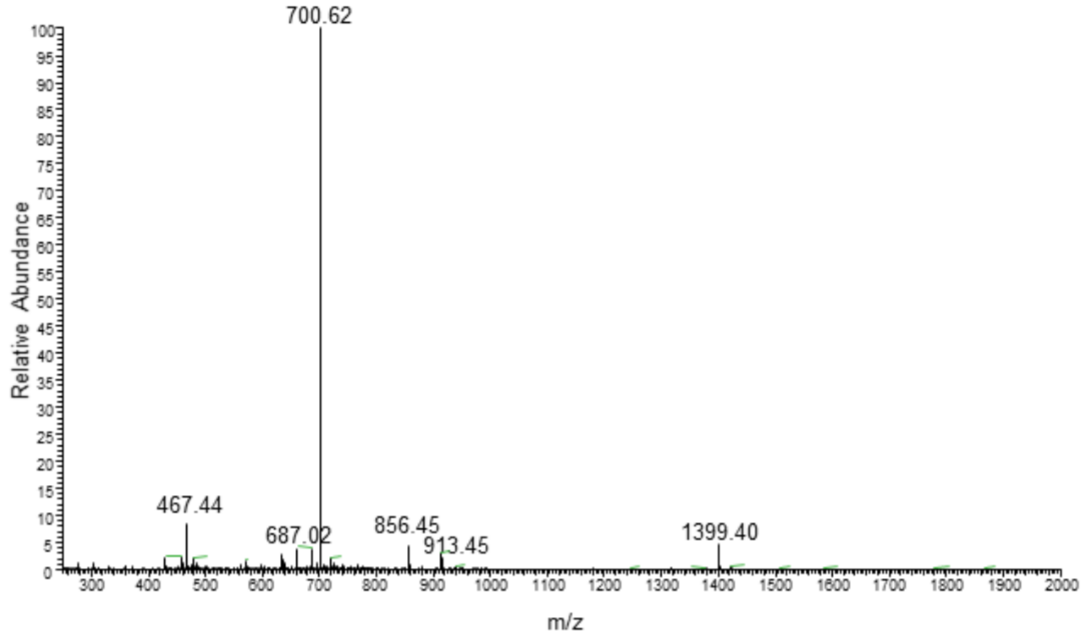

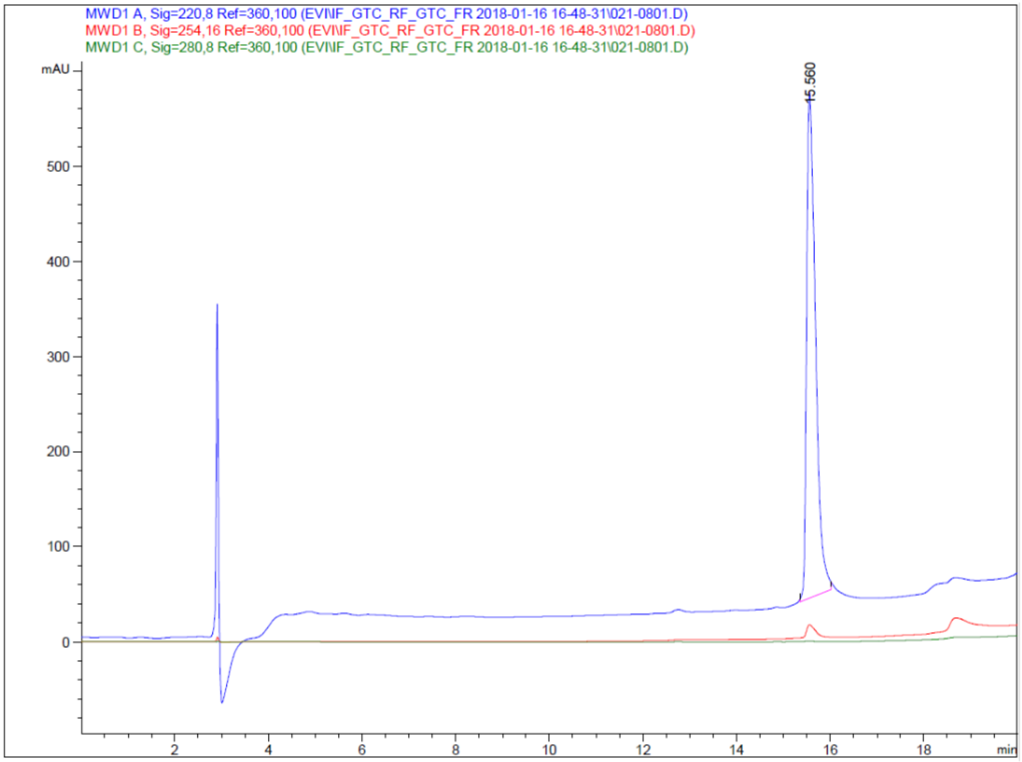

Figure S18. The MS spectra and the HPLC chromatogram of fYF (Column: Phenomenex Luna C18 (250 x 4.6 mm, particle size: 5 micron, pore size: 100 Å); Gradient: 5-80% 20min 1.2 mL min^{-1}).

Compound: **fIF**

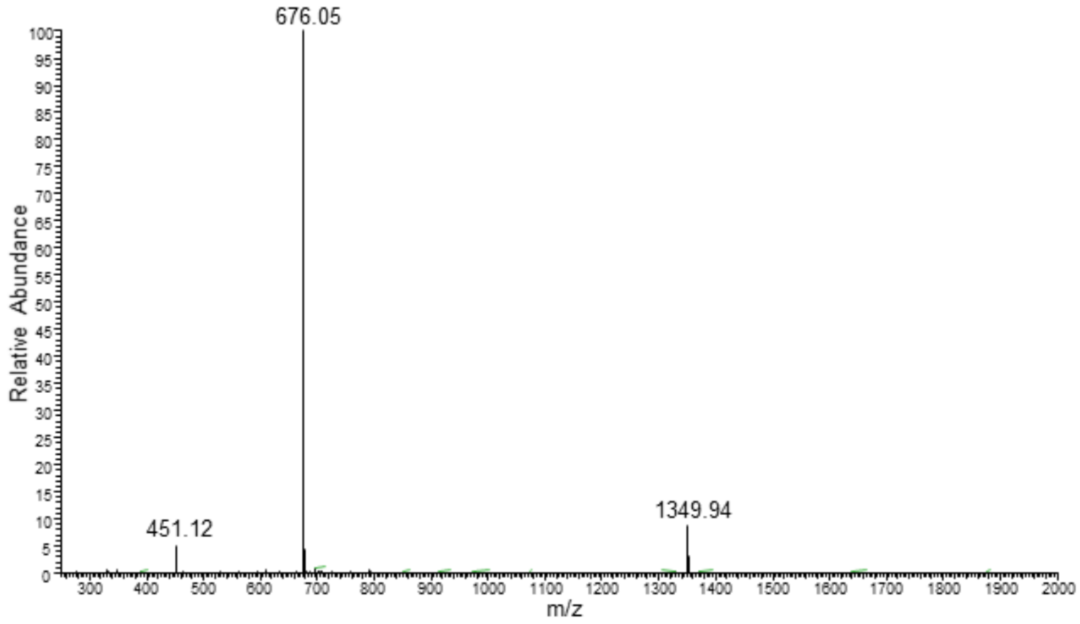

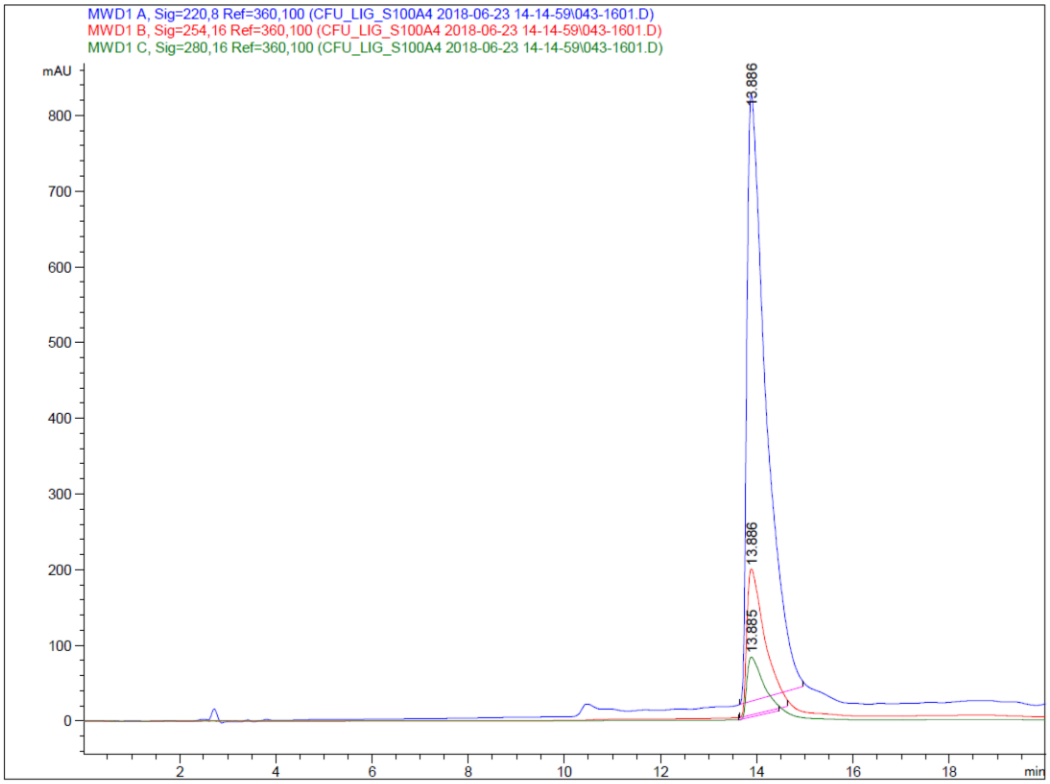

Figure S19. The MS spectra and the HPLC chromatogram of fIF (Column: Phenomenex Luna C18 (250 x 4.6 mm, particle size: 5 micron, pore size: 100 Å); Gradient: 40-60% 20min 1.2 mL min^{-1}).

Compound: **fTW**

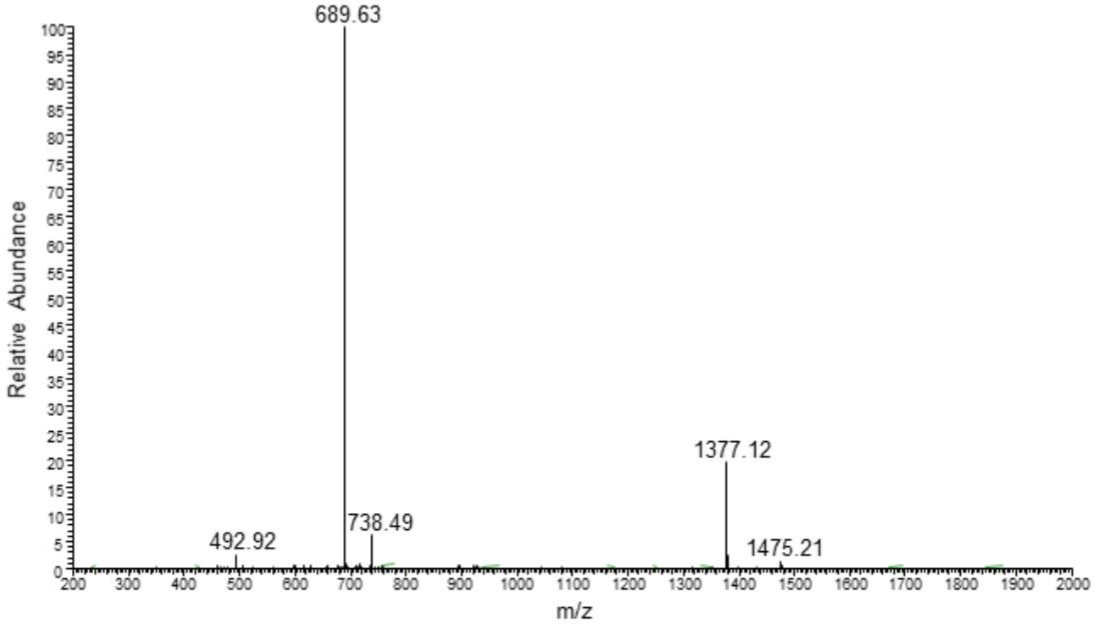

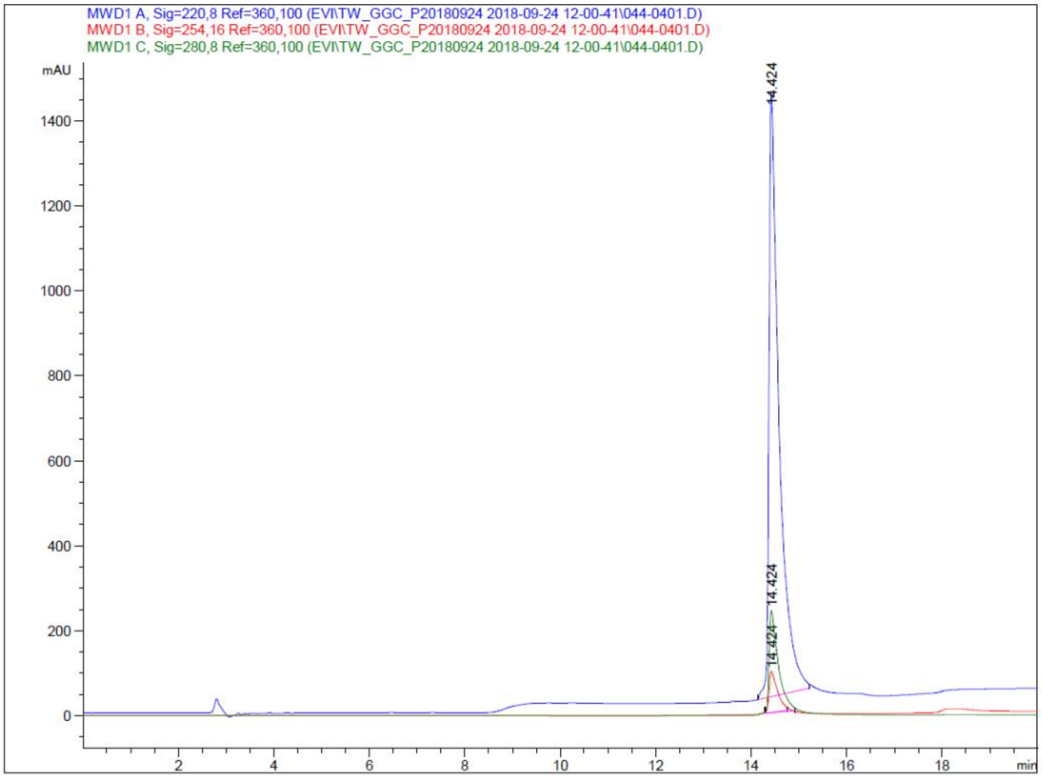

Figure S20. The MS spectra and the HPLC chromatogram of fTW (Column: Phenomenex Luna C18 (250 x 4.6 mm, particle size: 5 micron, pore size: 100 Å); Gradient: 5-80% 20min 1.2 mL min^{-1})

Compound: **fRF**

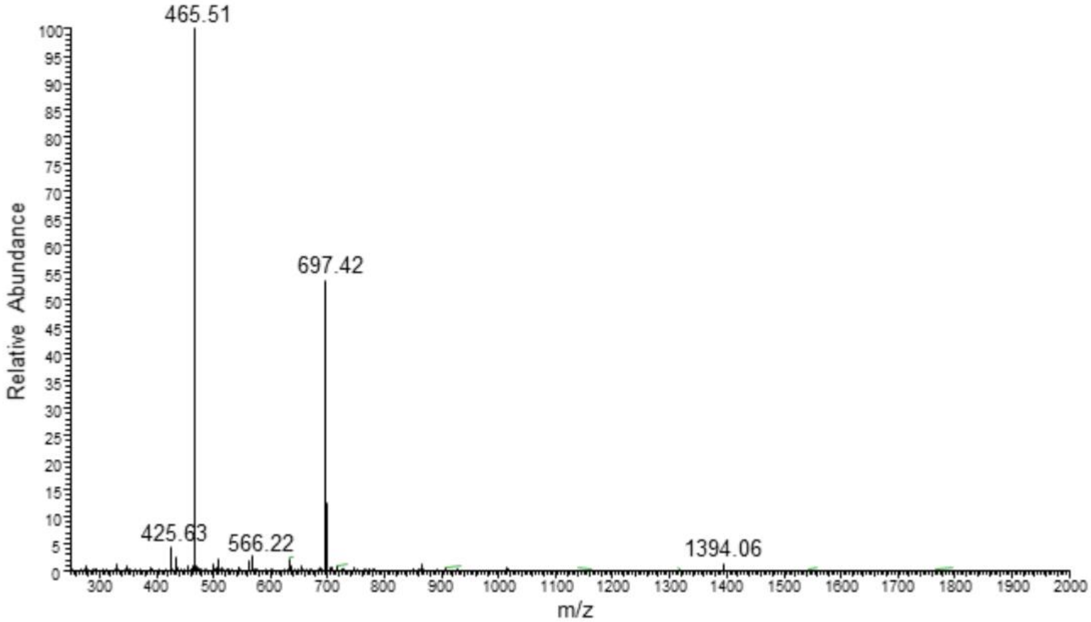

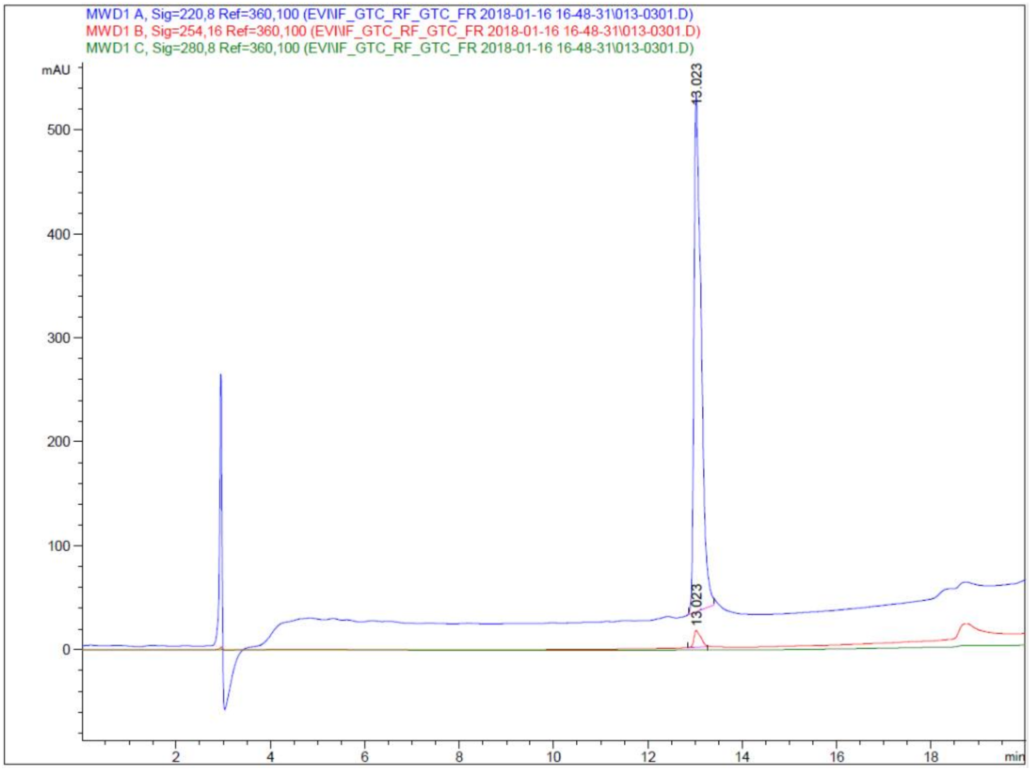

Figure S21. The MS spectra and the HPLC chromatogram of fRF (Column: Phenomenex Luna C18 (250 x 4.6 mm, particle size: 5 micron, pore size: 100 Å); Gradient: 5-80% 20min 1.2 mL min^{-1})

Compound: **fVL**

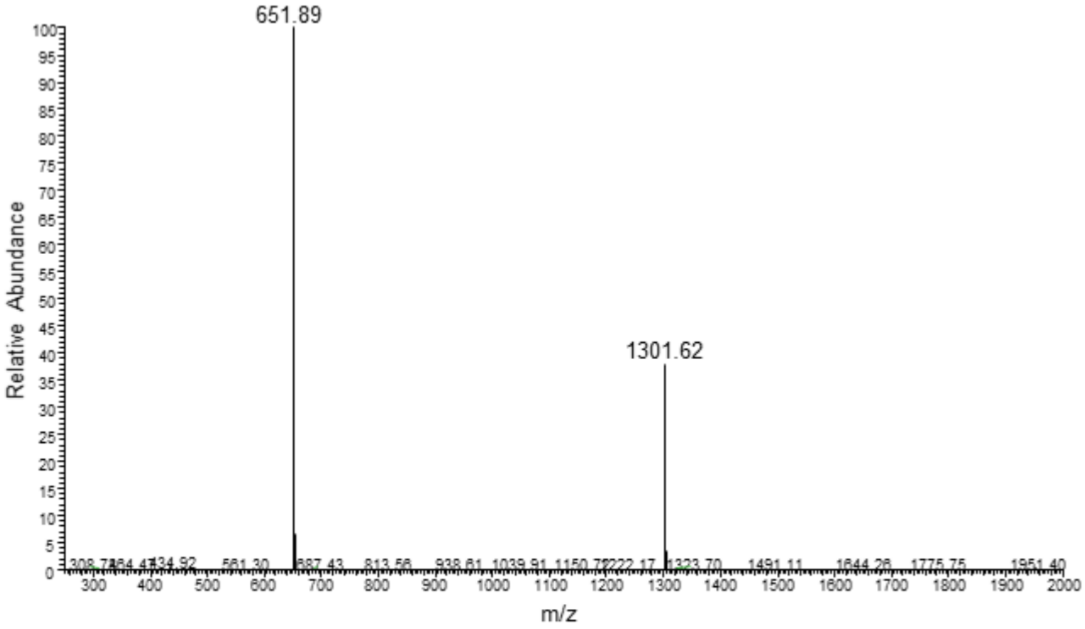

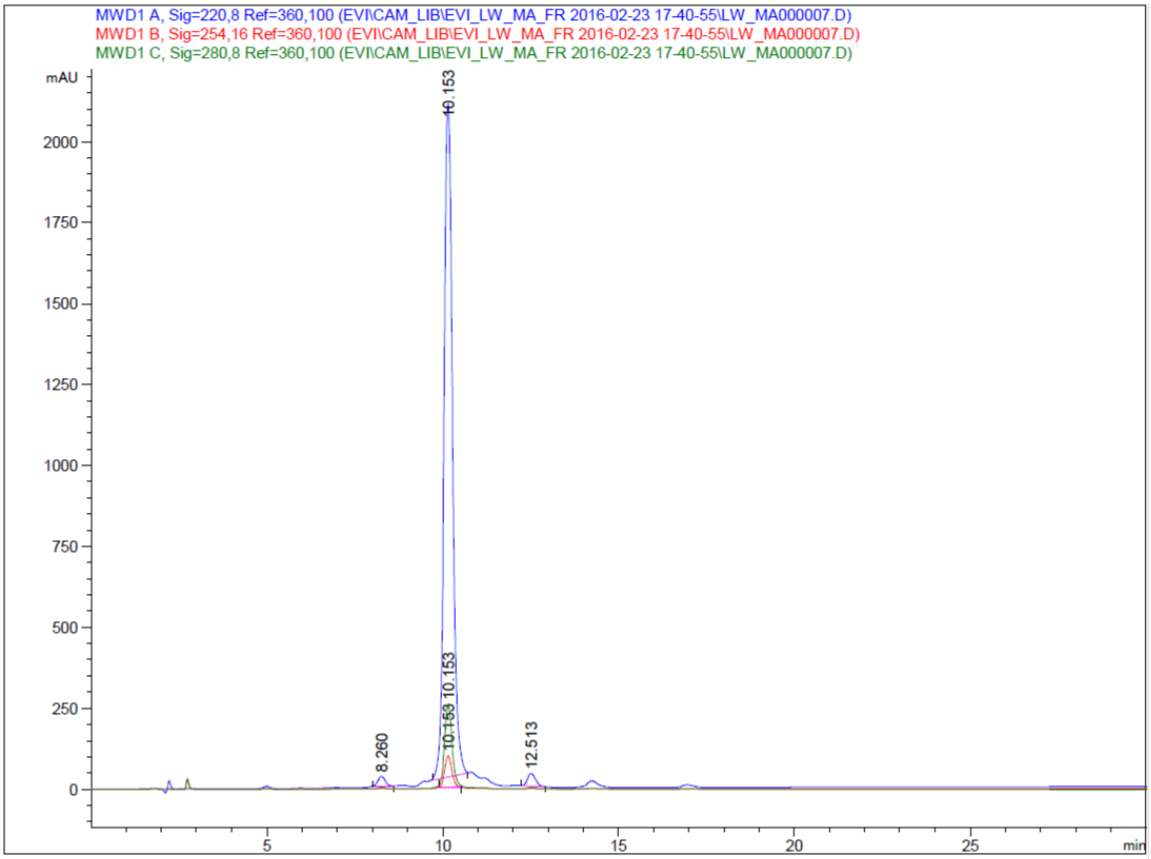

Figure S22. The MS spectra and the HPLC chromatogram of fVL (Column: Phenomenex Luna C18 (250 x 4.6 mm, particle size: 5 micron, pore size: 100 Å); Gradient: 40-70% 20min 1.2 mL min^{-1})

Compound: **fRR**

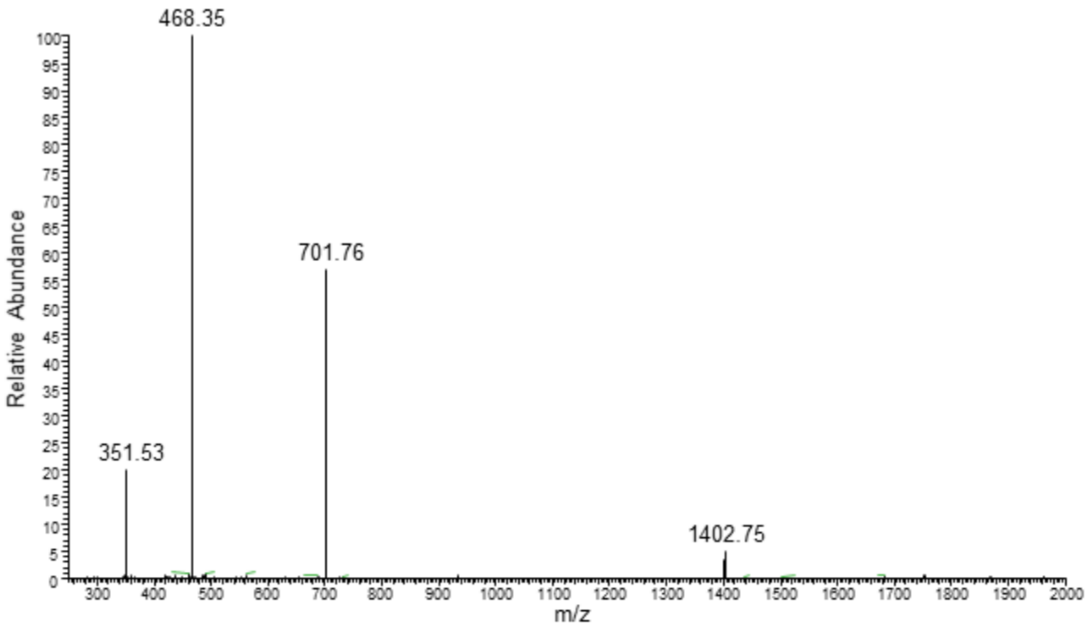

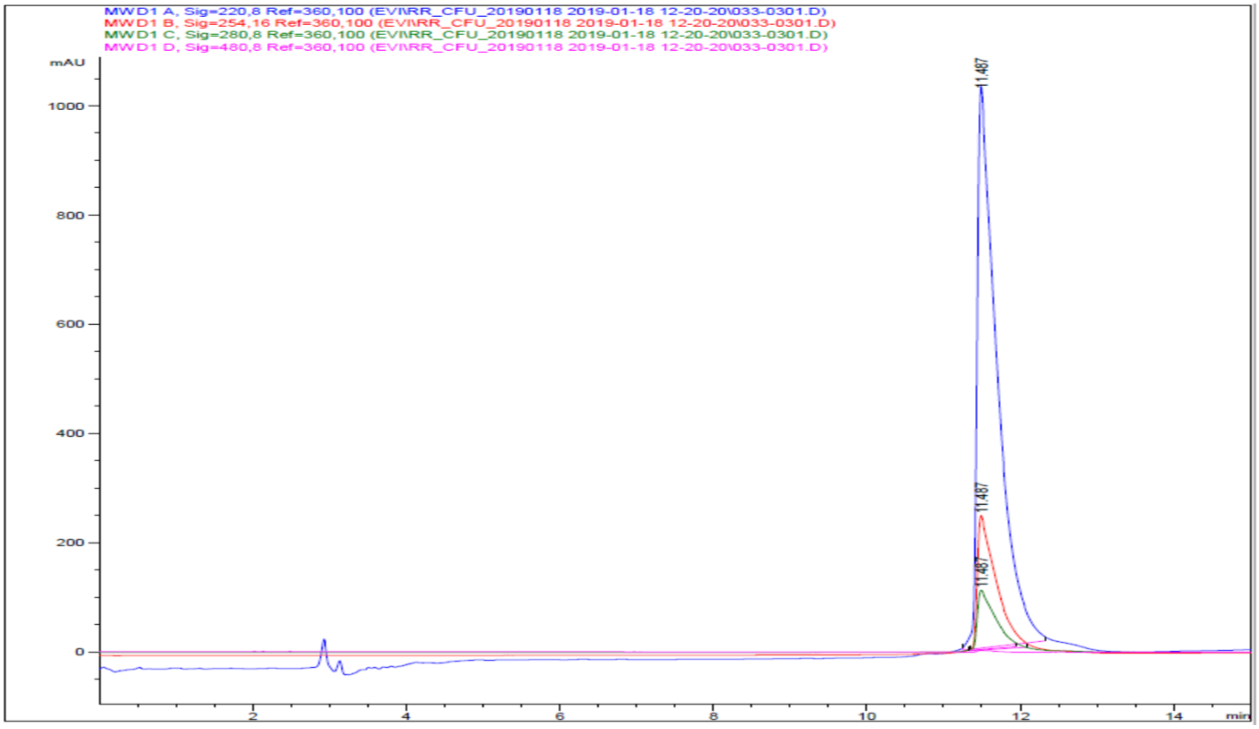

Figure S23. The MS spectra and the HPLC chromatogram of fRR (Column: Phenomenex Luna C18 (250 x 4.6 mm, particle size: 5 micron, pore size: 100 Å); Gradient: 5-80% 20min 1.2 mL min^{-1})

Compound: **fTI**

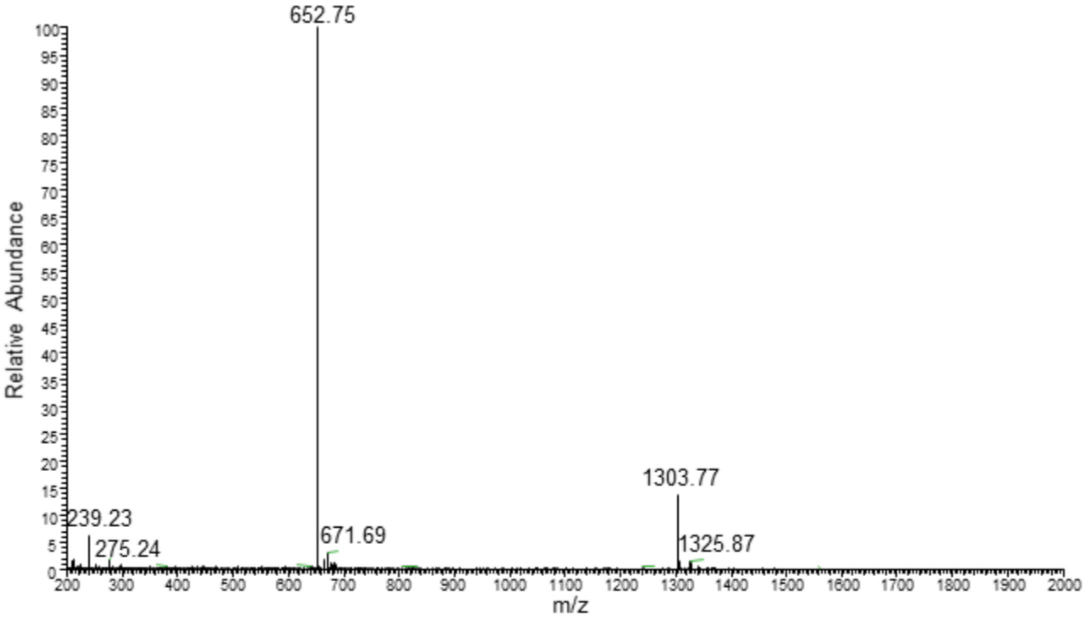

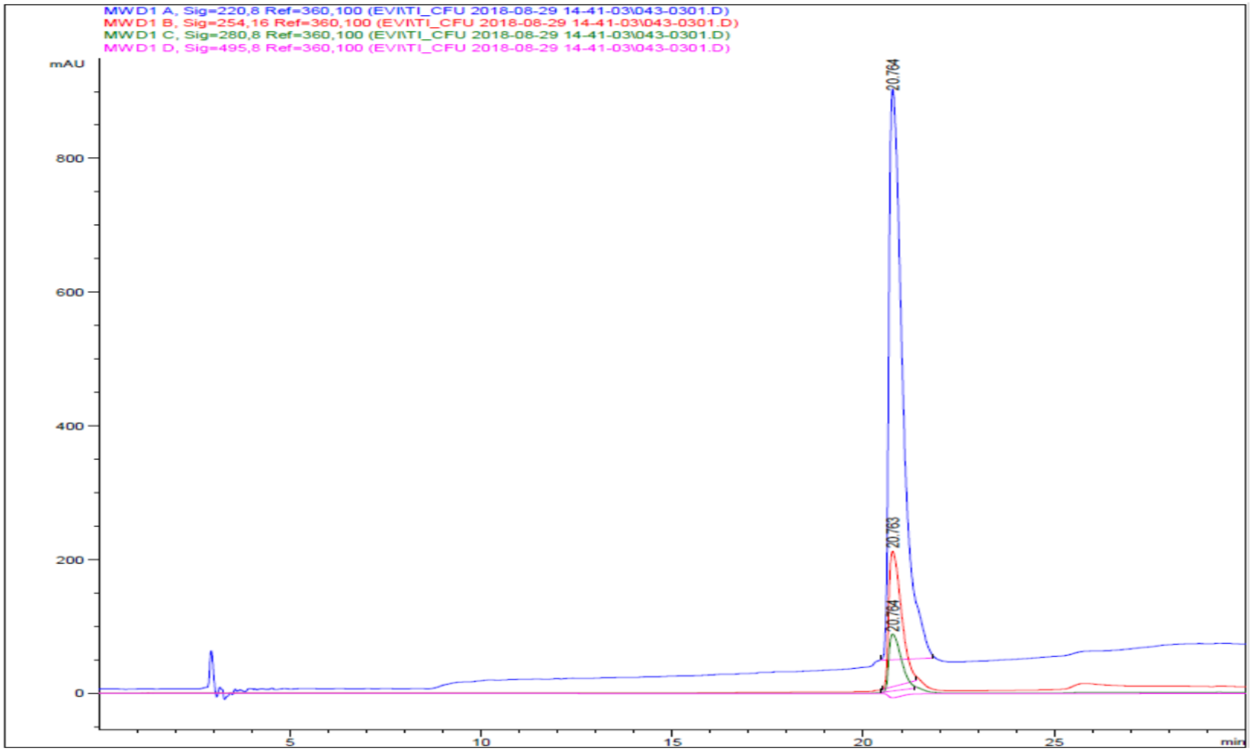

Figure S24. The MS spectra and the HPLC chromatogram of fTI (Column: Phenomenex Luna C18 (250 x 4.6 mm, particle size: 5 micron, pore size: 100 Å); Gradient: 5-80% 20min 1.2 mL min^{-1})

Compound: **fTM**

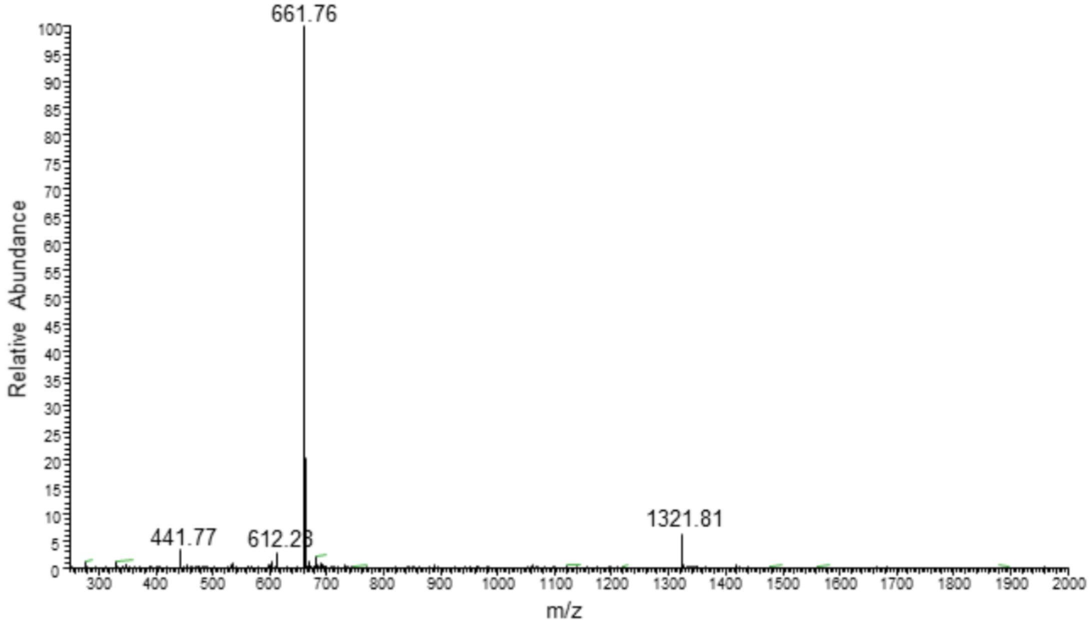

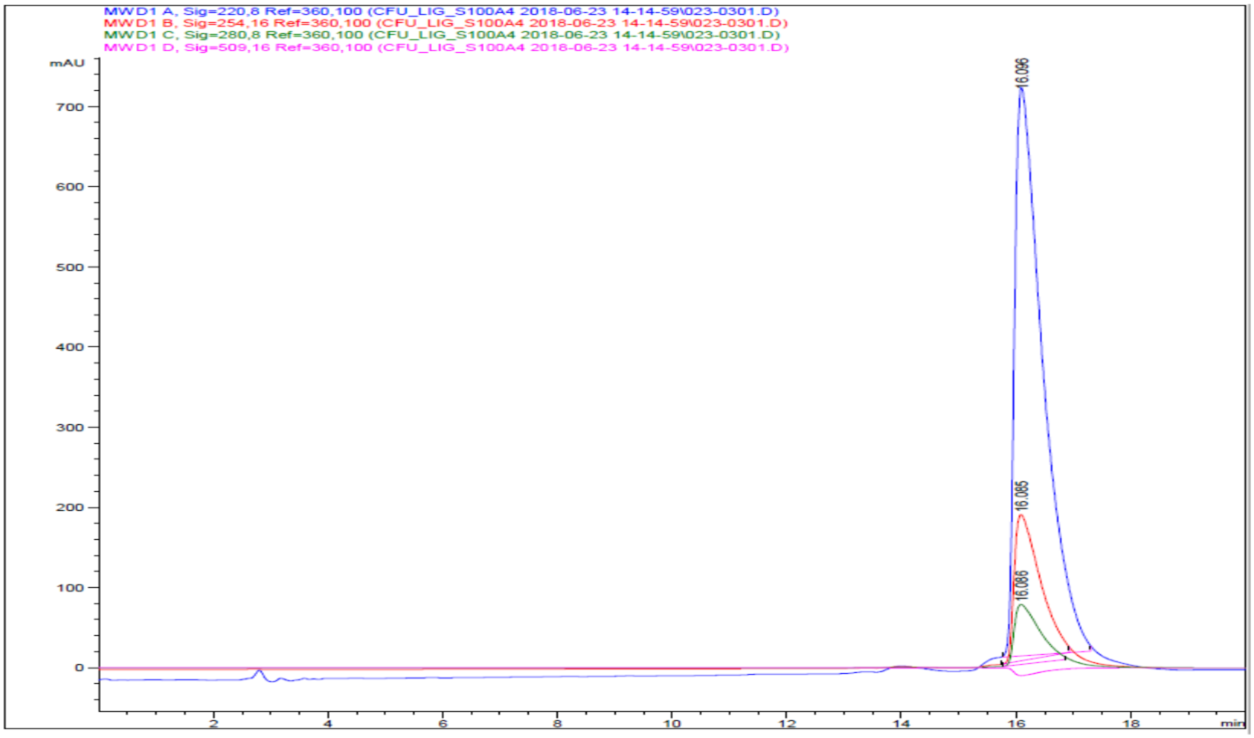

Figure S25. The MS spectra and the HPLC chromatogram of fTM (Column: Phenomenex Luna C18 (250 x 4.6 mm, particle size: 5 micron, pore size: 100 Å); Gradient: 30-50% 20min 1.2 mL min^{-1})

Compound: **fIL**

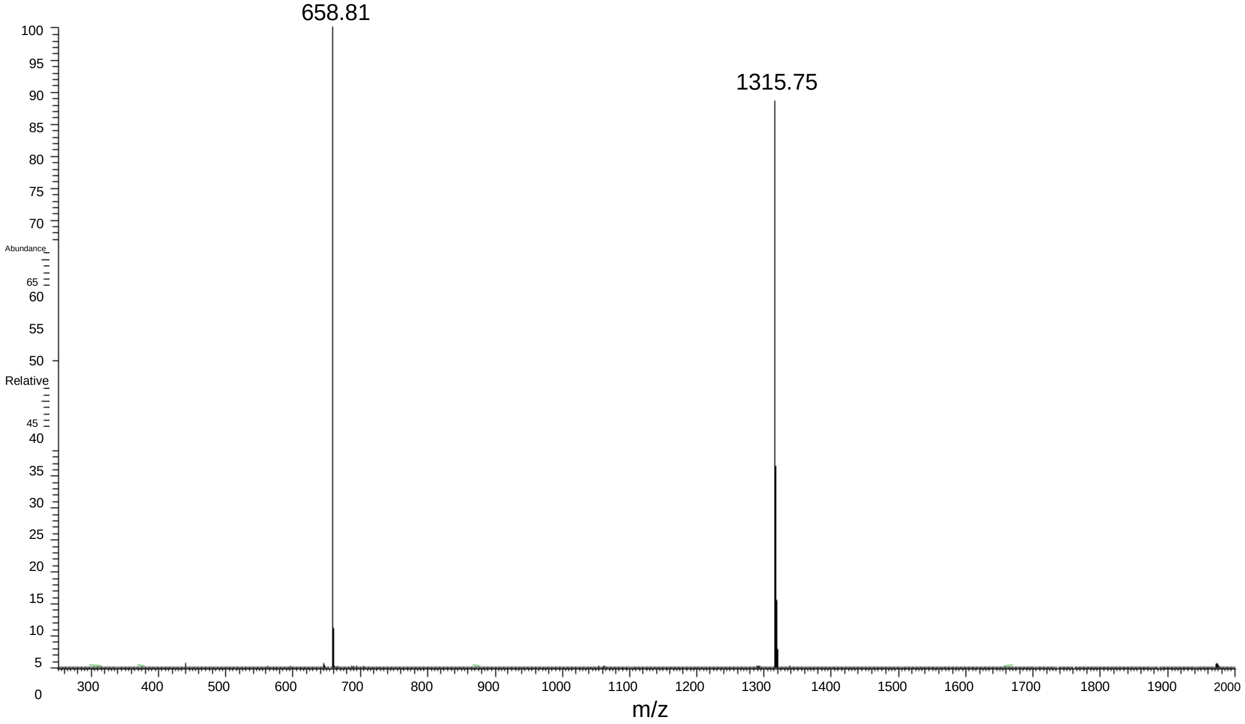

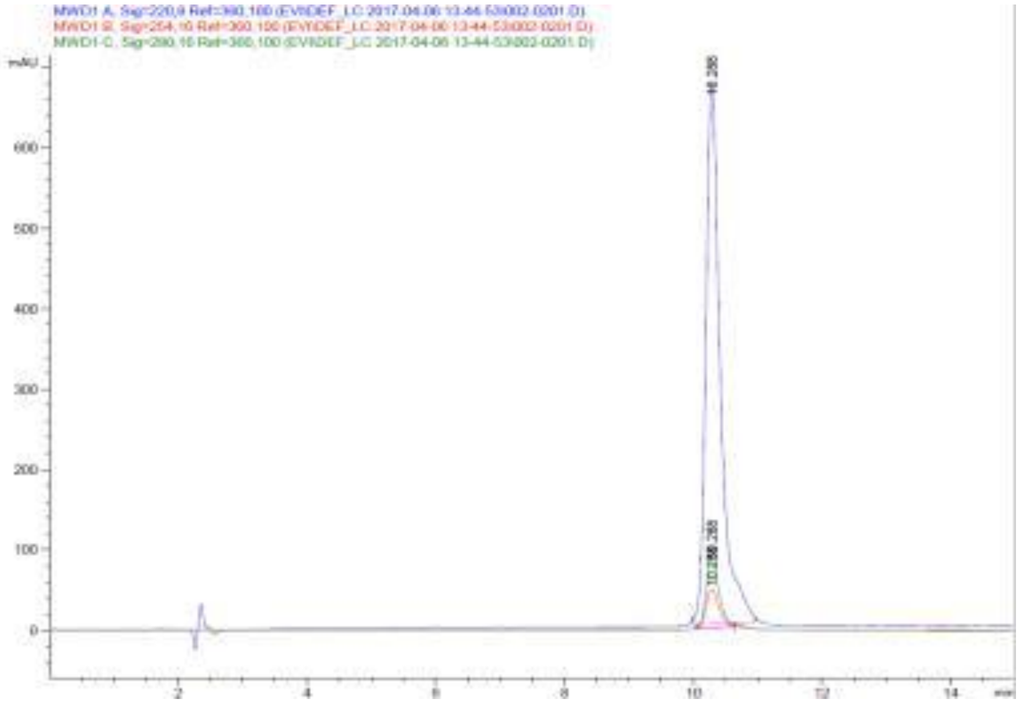

Figure S26. The MS spectra and the HPLC chromatogram of fIL (Column: Phenomenex Luna C18 (250 x 4.6 mm, particle size: 5 micron, pore size: 100 Å); Gradient: 40-70% 20min 1.2 mL min^{-1})
